# Exon Array Analysis using re-defined probe sets results in reliable identification of alternatively spliced genes in non-small cell lung cancer

**DOI:** 10.1186/1471-2164-11-676

**Published:** 2010-11-30

**Authors:** Wolfram Langer, Florian Sohler, Gabriele Leder, Georg Beckmann, Henrik Seidel, Jörn Gröne, Michael Hummel, Anette Sommer

**Affiliations:** 1 Bayer Schering Pharma AG, Global Drug Discovery (GDD) - Target Discovery, Müllerstrasse 178, 13342 Berlin, Germany; 2 Institute of Biochemistry, Justus-Liebig-University of Giessen, Heinrich-Buff-Ring 58, 35392 Giessen, Germany; 3 Dept. of General, Vascular and Thoracic Surgery, Charité - Universitätsmedizin Berlin, Campus Benjamin Franklin, Hindenburgdamm 30, 12200 Berlin, Germany; 4 Institute of Pathology, Charité - Universitätsmedizin Berlin, Campus Benjamin Franklin, Hindenburgdamm 30, 12200 Berlin, Germany

## Abstract

**Background:**

Treatment of non-small cell lung cancer with novel targeted therapies is a major unmet clinical need. Alternative splicing is a mechanism which generates diverse protein products and is of functional relevance in cancer.

**Results:**

In this study, a genome-wide analysis of the alteration of splicing patterns between lung cancer and normal lung tissue was performed. We generated an exon array data set derived from matched pairs of lung cancer and normal lung tissue including both the adenocarcinoma and the squamous cell carcinoma subtypes. An enhanced workflow was developed to reliably detect differential splicing in an exon array data set. In total, 330 genes were found to be differentially spliced in non-small cell lung cancer compared to normal lung tissue. Microarray findings were validated with independent laboratory methods for *CLSTN1*, *FN1*, *KIAA1217*, *MYO18A*, *NCOR2*, *NUMB*, *SLK*, *SYNE2*, *TPM1*, (in total, 10 events) and *ADD3*, which was analysed in depth. We achieved a high validation rate of 69%. Evidence was found that the activity of FOX2, the splicing factor shown to cause cancer-specific splicing patterns in breast and ovarian cancer, is not altered at the transcript level in several cancer types including lung cancer.

**Conclusions:**

This study demonstrates how alternatively spliced genes can reliably be identified in a cancer data set. Our findings underline that key processes of cancer progression in NSCLC are affected by alternative splicing, which can be exploited in the search for novel targeted therapies.

## Background

Lung cancer accounts for a quarter of all cancer mortalities in the U.S. [1]. Non-small-cell lung cancer (NSCLC) is a histologically defined sub-group that represents 75 to 80% of all lung cancer cases. NSCLC can be subdivided into adenocarcinoma (AdCa), squamous cell carcinoma (SCC), and large cell lung cancer (LCLC). The high mortality rate of lung cancer can be attributed to late diagnosis and thus an already metastasised and aggressive tumour. Platinum-based chemotherapy in combination with taxanes, camptothecins, or vinca alkaloids, is the first-line treatment of choice for patients with advanced NSCLC [2]. Yet, survival time is short and the five-year survival rate has only risen slightly since 1987 [1]. New therapies address molecular targets that are involved in tumour progression or angiogenesis (e.g. EGFR, VEGF-R) [3]. These involve small molecule drugs directed against mutated EGFR (e.g. gefitinib, erlotinib), as well as monoclonal antibodies directed against EGFR (cetuximab) and against VEGF (bevacizumab). These targeted therapeutics have provided some clinical benefit but also underlined that the molecular target needs to be accessible in the tumour type under treatment. That is, administering the drug will provide benefit to patients only for specific sub-types of the tumour where the target is relevant, thereby introducing the concept of an individualised therapy. Novel drugs for a targeted therapy that consider the sub-type specific tumour biology are urgently needed as treatment of NSCLC - a major unmet clinical need. In order to better understand molecular characteristics of NSCLC and derive novel molecular targets, studies have investigated gene expression in clinical samples [4] or in novel xenograft models derived from NSCLC specimens [5], gene expression under compound treatment (e.g. Sagopilone [6]), DNA copy-number variation [7], and epigenetic changes [8] in NSCLC in an unbiased approach. Yet, the changes caused by alternative splicing are relatively unexplored in NSCLC.

Alternative splicing (AS) describes the process by which pre-mRNA is spliced in different ways thus giving rise to distinct mature mRNA transcripts [9]. AS events can be characterised as inclusion or skipping of a complete exon (cassette exon, CE), prolongation or shortening of an exon (alternative 5'- or 3'-splice site), retention of an intron (IR), inclusion of only one exon from an array of two or more exons (mutually exclusive exons, MX), and alternative poly-A site [10]. Also alternative start of transcription can lead to different exon-exon junctions; however, this mechanism is not an AS event and its regulation need not be at the level of splicing. It has become evident that more than 73% of all human genes are alternatively spliced [11,12]. AS plays a major role in gene regulation, both in normal tissues as well as in disease. In cancer, AS has an impact on cellular processes related to tumour progression, including inhibition of apoptosis, tumour invasion, metastasis, and angiogenesis [13]. Changes of the AS pattern of a gene can be triggered by differential expression of splicing factors or by changes up-stream of the splicing machinery. One example is SRPK1, a kinase that is over-expressed in breast, colon, and pancreas carcinoma [14]. SRPK1 phosphorylates the splicing factor SF2/ASF, thereby mediating its import into the nucleus and recruitment to nuclear speckles [15]. This process affects the AS of multiple target genes (e.g. *BIN1*, *S6K1*, *MNK2*) which contributes to tumour progression [16]. Based on the analysis of ESTs from normal and cancerous tissues it was suggested that alterations affecting the splicing machinery and its regulation are a further hallmark of cancer progression [17]. Both the identification of RNA binding proteins affecting the AS pattern as well as their target sequences are active fields of research [18,19].

Most studies of AS in cancer focused on the analysis of individual genes. Several genes are well-known for AS and derived alternative proteins have different functionalities in tumour compared to normal tissue. One member of the Bcl-2 family is Bcl-X (*BCL2L1*), whose short transcript variant Bcl-X_*S *_promotes apoptosis. AS results in a longer exon in the transcript variant Bcl-X_*L *_in cancer cells. In contrast to the short transcript variant, this isoform has an anti-apoptotic function [20]. *CD44 *is another example of a gene that is affected by AS. Ten variant exons in this gene generate multiple transcript variants. In most tissues, the short isoform CD44_*S *_lacking all variant exons is expressed. Longer transcript variants containing one or many variant exons were found in specific cell types as well as in cancer cells. It was shown that the transcript variants of *CD44 *are involved in angiogenesis and metastasis [21,22]. AS can also yield new epitopes in tumour cell surface proteins or in proteins of the extracellular matrix of tumour cells that can be exploited for therapy via targeting by an immunoconjugate. Using such an approach, CD44-v6 was targeted by the immunoconjugate bivatuzumab mertansine [23]. The Fibronectin (*FN1*) gene codes for an extracellular matrix protein that contains three cassette exons, among them the extradomain B exon (EDB) [24]. An antibody fragment targeting the onco-foetal antigen FN1-EDB (L19-SIP) is currently in pre-clinical development [25]. All these examples demonstrate that AS is a highly interesting field: on the one hand, elucidating induced changes along the *hallmarks of cancer *[26] and, on the other hand, in the search for new drug targets, both for small molecule as well as for antibody-mediated approaches. Nevertheless, it remains a relatively unexplored area. Until recently, methods for a global analysis of AS were challenging and required a great deal of effort.

As a new technology that allow an unbiased analysis of AS, splice variant sensitive microarrays became commercially available in 2005. In this study, we utilise the oligonucleotide microarray Human Exon 1.0 ST Array (Affymetrix, Santa Clara, CA, USA). This array contains 6.5 million probes targeting known and predicted exons of the human transcriptome [27]. Probe sequences were designed in such a way that up to four probes compose a probe set that maps to one exon of a gene. Probe intensities can be summarised on the gene level which provides information about expression of the whole gene. In addition, the exon array technology allows summarisation per probe set which provides exon expression values. One can determine the relative inclusion or skipping rate of an exon between two or more sample groups (*differential splicing*) using both metrics together. Although alternative start of transcription is not an AS event, the exon array technology can also detect differences in the usage of transcription start sites. In the following, we also consider this kind of mechanism when speaking of *differential splicing*.

In previous studies (review [28]), the exon array technology was used to detect differences in AS patterns between healthy human tissues [29], between human populations [30], under hypoxia conditions [31], and in disease tissues. Differential splicing was analysed in several types of cancer, among them colon, breast, prostate, bladder, and head and neck cancers [27,32,33]. Clinical samples of NSCLC have been investigated in two studies: Xi et al. analysed a data set of matched pairs of AdCa [34]. They found evidence for differential splicing in 2369 genes and further analysed a subset of 729 genes that are cancer-related according to pathway annotations. Of 11 genes selected for a validation using independent laboratory methods, differential splicing was confirmed in six genes (*CEACAM1*, *ERG*, *RASIP1*, *VEGFC*, *CDKN2A*, *CDH3*). Lin et al. analysed differential splicing in a large data set consisting of samples of AdCa and SCC of NSCLC besides colon and breast cancer [35]. This data set does not contain samples of healthy tissue for comparison.

Data analysis of exon arrays remains a challenging task despite several different approaches described recently [27,33-35]. In the workflow proposed by Affymetrix, exon level expression values are normalised to gene level expression values to calculate the *splicing index *(SI) [36]. Differentially spliced genes are identified using both the magnitude of change (SI) as well as significance, e.g. p value obtained from a Student's t-test. It became evident that the standard workflow leads to a high false positive rate which especially affects noisy data sets such as cancer data sets with high intrinsic variability due to inter-patient heterogeneity [27]. Three sources of artefacts are thought to be the major cause of false positives: (1) Probe intensities at the background noise, (2) cross-hybridising probes, and (3) imprecise calculation of the SI.

(1) Probes corresponding to exons that are not expressed in a particular sample group measure the background noise and thus will not be informative. Still, their expression value does not follow the overall gene expression level thus leading to high SI values and false positive results. It is generally accepted that it is desirable to remove this kind of probes before conducting the analysis [37]. Here, the difficulty resides in identifying probes that are in fact detecting signals above the background noise. Algorithms initially employed like DABG were based on the GC-content of the probes [29,38]. Recent advances in this field incorporate a statistical model based on probe sequences (MAT algorithm [39]) or a thermodynamic model of oligonucleotide hybridisation (MSNS algorithm [40]).

(2) Cross-hybridising probes are probes that bind other sequences besides the intended exon. This can lead to a constantly high expression value that does not follow the gene-level expression value. Again, this will lead to a high SI and to false positives. To date, this issue has been addressed with either of two approaches: mapping of probes sequences to the transcriptome and flagging potential candidates probes that have multiple hits [41] or filtering out probes with a constantly high expression value [36].

(3) The third kind of artefact can be attributed to difficulties in measuring the SI. As mentioned by Affymetrix, determination of the correct gene level summarisation value can be cumbersome [36]. It can be estimated in general more reliably for a gene with many constitutive exons and preferably only a small number of exons affected by AS. A number of improvements were published trying to identify constitutive exons [42-44]. As another approach, Shah and Pallas used a correlation-based approach in favour of calculating the SI [45]. Recently, Möller-Levet et al. introduced the new metric VFC which is a weighted fold-change based on probe set reliability, i.e. detection above background score [33].

In addition to the identification of artefacts as mentioned above, a method of analysis needs to consider the relationship between exons and genes and also annotations of known transcript variants. Apparently, probe set definitions and annotations provided by Affymetrix were used in many studies. Affymetrix continuously updates the annotation files, i.e. the assignment of probe sets to the latest set of genes and mRNA sequences. Yet other information is not updated, such as the assignment of probes to probe sets (chip definition), the assignment of probe sets to a putative gene locus (transcript cluster), and the reliability assignment of a probe set (core set, extended set, full set). As this information is not static, an analytical method would benefit from an update of all of these definitions: a probe looking perfect at design time might in fact map to multiple targets suggesting exclusion from a probe set. Unfortunately, transcript and gene annotations may contain some errors; corrections might require remapping between probe sets and the new gene locus/loci. Predicted exons can get backed by transcript evidence but might still be omitted from a study since they have not been moved to the core set. In part, these issues have been addressed with the database X:Map that provides an up-to-date mapping of probe sequences to transcripts and genes [46]. In some studies, X:Map is used either directly or indirectly via the Bioconductor package Exonmap [47,48]. All of the analysis workflows discussed above underline that analysing an exon array data set is a multi-faceted challenge. In this study, we propose a new workflow that addresses the above issues. We generated a new chip definition that represents an updated composition of probe sets and their assignment to known transcripts and genes. Furthermore, we use advanced algorithms in order to remove artefacts and to detect differentially spliced genes.

With the new workflow we identified genes that exhibit differential splicing in NSCLC compared to normal adjacent lung tissue (NAT). In order to extend our biological understanding of AS in cancer, alterations of splicing patterns between NSCLC and NAT were analysed on a genome-wide level. We generated an exon array data set derived from matched pairs of NSCLC and NAT including both the AdCa and the SCC subtype. Initially, we analysed this data set with a *standard workflow *based on the SI (workflow proposed by Affymetrix [49]) and the generally accepted analysis of variance (ANOVA; workflow implemented in Partek^® ^Genomics Suite). Several improvement steps led to our *enhanced workflow*. After using the final version, genes that are known to be differentially spliced in NSCLC versus NAT can be found ranking highly in the result list (e.g. *FN1*). In total, 14 genes of this result list were selected for validation using independent laboratory methods. We succeeded in validating 69% of all differential splicing events. This includes ten events that are genuine AS events and one alternative transcription start site event. This proves that our enhanced workflow can reliably identify genes that are affected by AS even in a clinical cancer data set that contains different subtypes of NSCLC and that reveals a high heterogeneity between the patients. We also examined the data set for genes that exhibit a different splicing pattern between two subtypes of NSCLC (AdCa versus SCC).

## Methods

### Data set and specimens

All investigations were performed in accordance with the Declaration of Helsinki. After informed consent had been obtained, biopsies of tumour tissue and of healthy mucosa from patients with lung cancer undergoing elective curative surgery from 2001 to 2003 at the Department of Surgery, Charité, Campus Benjamin Franklin, Berlin, Germany, were collected by the clinical partner with approval by the local ethics committee (reference number EA4/006/05). Cancer and corresponding epithelial normal tissue specimens were snap frozen in liquid nitrogen within 20 min following excision and stored at -80°C. All tissue samples were evaluated by a pathologist before and during macrodissection to ensure an enrichment of vital tumour cells and normal epithelial of 80 - 90%. Total RNA preparation was performed with the RNeasy^® ^kit (Qiagen, Hilden, Germany). In addition, total RNA isolated from clinical samples was obtained from Cambridge Biosciences, Ltd. (Cambridge, UK). In total, 11 paired samples of adenocarcinoma of NSCLC and NAT (*n *= 22) and seven paired samples of squamous cell lung carcinoma and NAT (*n *= 14) were analysed (for details see additional file 1). All NSCLC samples have at least 60% tumour content or were enriched by macrodissection. Concentration of total RNA was quantified using a NanoDrop spectrometer and quality of RNA was measured on an Agilent Bioanalyzer (Agilent RNA 6000 Pico and Nano Kit; Agilent Technologies, Santa Clara, CA, USA). All samples had an RIN (RNA integrity number) ≥ 6.9.

### Synthesis of fragmented cDNA and microarray hybridisation

Sample amplification and preparation for microarray hybridisation was performed according to the GeneChip^® ^whole transcript sense target labelling assay (manual rev. 4, Affymetrix). In brief, in this procedure 2 *μg *total RNA of each sample were depleted from rRNA (RiboMinus™ Transcriptome Isolation Kit (Human/Mouse), Invitrogen, Carlsbad, CA, USA), reverse transcribed to cDNA, amplified by in vitro transcription, and reverse transcribed to cDNA. Fragments between 40 and 70 bp were generated enzymatically from 5.5 *μg *cDNA, labelled terminally, hybridised onto the Affymetrix Human Exon 1.0 ST array, stained and washed, and finally scanned using an Affymetrix GeneChip^® ^Scanner 3000 7G. Quality of scanned array images was assessed visually using GeneChip^® ^operating software (GCOS). The raw data (CEL-files) have been deposited in the ArrayExpress Archive under accession number E-MEXP-2644.

### Pre-processing and data analysis

In the analysis of the exon array data, we included only probe sets derived from the *core set*. These probe sets correspond to exons that are part of well-annotated transcripts, e.g. RefSeq transcripts [27]. However, in addition, the exon array contains probe sets that are backed by less evidence such as EST data and Genscan predictions, and thus we omitted these probe sets. All pre-processing operations were conducted in Partek^® ^Genomics Suite version 6.5 (Partek Inc., St. Louis, MO, USA). Probe intensities were quantile normalised, a background adjustment was performed as implemented in Partek^® ^Genomics Suite using a model based on the probe sequence, and finally probe intensities were summarised using the RMA algorithm. All probe set intensities were normalised using quantile normalisation. For quality assurance and in order to detect outlier samples, the overall gene expression was analysed by means of a principal component analysis (PCA) and with hierarchical clustering based on Pearson's correlation (average linkage). Quality assurance with PCA and hierarchical clustering was also performed using gene level expression values. To this end, probe set expression values were summarised to gene level expression values using Tukey's biweight algorithm as implemented in Partek^® ^Genomics Suite.

We omitted all genes containing fewer than 5 probe sets (leaving 277182 probe sets that correspond to 16613 genes). Analysis of variance (ANOVA) for differential splicing was performed as implemented in Partek^® ^Genomics Suite (alternative splicing ANOVA). For each gene, a mixed linear model (MLM) including the factor pathology group *G *(tumour or NAT), the random factor patient *P*, and the factor exon *E *was fitted (gene MLM; equation 1). An ANOVA on the effects alternative splicing (E * *G*), pathology group (*G*), and patient (*P*) provides p values for the respective effect. As correction for multiple testing, the false discovery rate (FDR) was estimated after Benjamini-Hochberg. For each probe set, an MLM including the factors pathology group *G *and patient *P *was fitted (probe set MLM; equation 2). Expression ratios were obtained as the *contrast *of the pathology group (*G*) effect between tumour and NAT from the respective MLM.

(1) I=μ+G+P+E+E ∗ G+S(G∗P)+ϵ

(2) I=μ+G+P+ϵ

where *μ *is the overall mean of all probe set intensities *I *per gene or probe set, respectively, *S *is a sample-to-sample random factor nested in pathology group (*G*) and patient (*P*), and *ε *is the error term. The splicing index (SI) is defined as

(3) $$SI=\frac{{\text{cg}}_{\text{exon level}}}{{\text{cg}}_{\text{gene level}}}$$

where cg is the contrast of the pathology group (*G*) effect between tumour and NAT derived from the respective MLM.

To discover tumour sub-type dependent differences in the alternative splicing patterns, the levels of the factors pathology group *G *were changed from tumour and NAT to AdCa, SCC, and NAT. Expression ratios were obtained as the contrast of the pathology group (*G*) effect between tumour and NAT from a MLM ANOVA based on samples of each subtype, respectively. Likewise, SI values were calculated for each subtype. Information about the location of alternative splicing events identified from well-known transcript variants was obtained from UCSC genome browser (database hg19, table *knownAlt*, table schema described under http://genome.ucsc.edu/cgi-bin/hgTables?db=hg19&hgta_group=genes&hgta_track=knownAlt&hgta_table=knownAlt&hgta_doSchema=describe+table+schema, data available for download under http://hgdownload.cse.ucsc.edu/goldenPath/hg19/database/knownAlt.txt.gz).

### Background filter

Probe sets with a signal near the background noise are termed *absent*. We estimated the expression above background noise using the MAT algorithm [39] and utilised the implementation of MAT in ProbeEffects version 2.0.1. The software was modified in such a way that detection above background p values are generated per probe set instead of per gene as in the original implementation. The MAT background model is fitted per sample to genomic and anti-genomic background probes (GC-bins). Probe sets are considered to measure a signal above the background if *p *≤ 0.01. A probe set is treated as *present *if *p *≤ 0.01 in at least 75% of the samples of the respective pathology group (tumour or NAT). Otherwise, a probe set is treated as *absent *in the respective sample group. Only probe sets that are absent in both sample groups, i.e. absent both in tumour as well as in NAT, are filtered out. Genes containing fewer than five present probe sets are also removed.

### Re-definition of probe sets

New *core *level probe sets were defined using most up-to-date transcript data. From publicly available sources (Ensembl and Vega version 56.37a, RefSeq release 38), in total 269611 transcript variants related to 42429 gene loci were obtained. Non-redundant exons of all transcript variants were collected.

Overlapping exons cause splitting of such exons into distinct probe selection regions (PSRs). In contrast to the approach followed by Affymetrix, we do not include less reliable EST data and Genscan predictions for the generation of PSRs. Thus, we try to avoid extensive fragmentation of exons into small PSRs. It can be expected that PSRs will be larger and that each will be covered by more probes than in the Affymetrix probe set definition. Mapping information of exon array probes to the genome was obtained from X:Map version 56.37a [46]. Probe sets were created for PSRs that are covered by at least one probe. Probes mapping to multiple locations on the genome as well as PSRs from repetitive regions were filtered out (RepBase version 9.11, RM database version 20050112 [50]). The new probe set definition together with annotation are available as additional file 2.

### Laboratory validation of exon array results

Of each RNA sample, 50 *ng *total RNA were reverse transcribed in cDNA and amplified linearly according to manufacturer's instructions (WT-Ovation™ RNA Amplification System, NuGen Technologies, Inc., San Carlos, CA, USA). Endpoint reverse transcription PCR (RT-PCR) was carried out for AdCa/NAT paired samples in 20 *μl *reactions starting from 100 *ng *NuGen amplified template cDNA. Amplification using Immolase™ DNA polymerase (ImmoMix and ImmoMix Red, Bioline GmbH, Luckenwalde, Germany) was performed for 30 cycles at 60°C annealing temperature. Assays were designed to yield two distinct amplicons, one of smaller size skipping the cassette exon, the larger one including the enframed cassette exon (see additional file 3). HPLC purified primers were obtained from TIB MOLBIOL GmbH (Berlin, Germany). PCR products were separated by electrophoresis using MetaPhor^® ^Agarose (Lonza, Basel, Switzerland), cut out of the gel, and extracted (QIAEX^® ^II Gel Extraction Kit, Qiagen, Hilden, Germany). The extracted products were ligated into a TOPO^® ^pCR4^® ^vector (TOPO TA Cloning^® ^Kit For Sequencing, Invitrogen, Carlsbad, CA, USA) and transferred into chemically competent TOP10 cells. Plasmid DNA was isolated and sequenced with M13-reverse and M13(-20)-forward primers at AGOWA GmbH (Berlin, Germany). Subsequently, the PCR amplicon sequences were assembled and checked against the transcript sequences annotated in Ensembl 56.37a.

Transcript variants were quantified using quantitative reverse transcription PCR (qRT-PCR) in paired samples of AdCa or SCC and NAT. qRT-PCR assays were designed using the Universal Probe Library Assay Design Center (Roche Diagnostics GmbH, Mannheim, Germany; https://qpcr.probefinder.com/) or the software Primer3 [51]. HPLC purified primers were obtained from TIB MolBiol GmbH. Assays were designed either to yield transcript variant-specific amplicons (transcript variant expression) or to yield amplicons in exons common to all known transcripts (gene level expression). Assay details are given in additional file 4. Where possible, assays were designed to span an exon-exon junction such that contaminations of genomic DNA have no influence. qRT-PCR was carried out in 10 *μl *reactions starting from 10 *ng *NuGen amplified template cDNA. Amplification was performed with MESA BLUE qPCR MasterMix Plus for SYBR^® ^Assays (Eurogentec, Seraing, Belgium). All qRT-PCR reactions were carried out in an Applied Biosystems ABI Prism^® ^7900 HT cycler with 40 cycles at 60°C annealing temperature. A dissociation curve was recorded after the run. All assays were checked with regard to efficiency and PCR product size using cDNA from cell lines. After each run, obvious outliers (no amplification, multiple products) were manually excluded from the subsequent analysis. *Ct *values were set in the logarithmic phase of the PCR-reaction. Averaging of technical replicates and normalisation to the geometric mean [52] of the endogenous control gene *Ct *values (ESD, POLR2A [53]) results in Δ*Ct *values. Both fold-changes (FC) as well as splicing index (SI) values were calculated from ΔΔ*Ct *values (see equations 5 and 6). Significance was determined by means of a paired t-test on the *SI *values.

(4) $$\Delta \Delta Ct=\Delta Ct_{\text{Tumour}}-\Delta Ct_{\text{NAT}}$$

(5) $$FC:=2^{-\Delta \Delta Ct}$$

(6) $$SI:=2^{-(\Delta \Delta Ct_{\text{transcript}\;\;\text{variant}}-\Delta \Delta Ct_{\text{gene level}})}$$

We consider exon array results to be validated successfully if qRT-PCR results show a significant (paired t-test *p *≤ 0.05) or high (*SI *≥ 2.0 or *SI *≤ 2.0^-1^) difference of transcript variant expression that is in agreement with our hypothesis of AS and the exon array results. Validation is successful if qRT-PCR results fulfil these criteria for at least one subtype of NSCLC (AdCa or SCC). In addition, if an RT-PCR result is available, it must exhibit the AS pattern in the majority of the samples analysed.

### Expression of FOX-genes

Gene expression levels of FOX1 (*A2BP1*) and FOX2 (*RBM9*) in different types of cancer and healthy body tissues were obtained from a collection of a total of 1015 samples hybridised on the Affymetrix GeneChip^® ^array HG-U133_Plus_2.0. Signal intensities were summarised and normalised with the MAS5.0 algorithm [54]. Mean values and standard deviations per sample group were calculated with log-transformed probe set intensities.

Expression of FOX1 and FOX2 transcripts was detected with RT-PCR as described above. Primer sequences were: FOX1, forward 5'-CCAGTTGTGGGTGCAGTCTA-3' and reverse 5'-AGCTTCCTTTCTCCCCACAT-3'; FOX2, forward 5'-GCGGACAGTATATGGTGCAGT-3' and reverse 5'-GGTTGCAGTAGCAGGCTGTG-3'. Alternatively spliced exons in RT-PCR products of FOX2 were identified after cleavage with exon specific restriction enzymes (FastDigest^® ^Hpy8I, AfeI, and HinfI; Fermentas, Glen Burnie, MD, USA). DNA fragments obtained from RT-PCR and optionally after digestion with restriction enzymes were separated by agarose gel electrophoresis as described above or using the Agilent Bioanalyzer (Agilent DNA 1000 Kit; Agilent Technologies, Santa Clara, CA, USA).

FOX1 gene expression, FOX2 gene expression, and FOX2 transcript variant expression was quantified using qRT-PCR as described above. Primers were: FOX1 gene level, forward 5'-CCTTACCTTCCTGGACTGATTC-3' and reverse 5'-GTAAGGCTGAGCCATTGTGTC-3'; FOX2 gene level, forward 5'-TGGAAATTAAGCCCAGTAGTTG-3' and reverse 5'-TGATACCCCCTCTTCCTGA-3'; FOX2 transcript variants, common forward primer 5'-GCGGACAGTATATGGTGCAGT-3'; FOX2 cassette exon, reverse primer 5'-TAGAGGTCAGCACCGTAAAATCC-3'; FOX2 exon skipping, reverse junction primer 5'-CATATCCACCCCTGGATAGG-3'.

## Results

**Figure 1:**
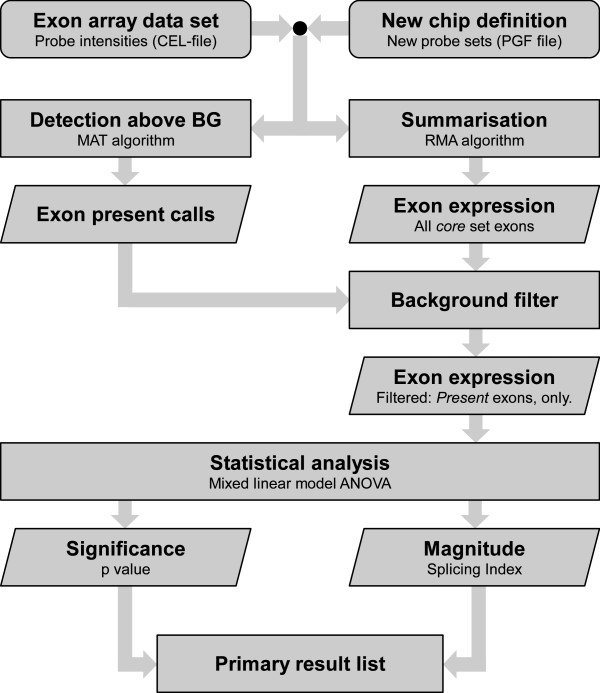
***Enhanced workflow *for the detection of genes that are affected by differential splicing**. A new definition of *core set *probe sets is the basis of the improved workflow. All probe intensities are summarised to exon expression levels. Estimation of detection above background noise leads to exon present calls. Exons that are *absent *in both sample groups will be removed by the background filter. In the statistical analysis, only genes with at least five *present *probe sets are considered. Both significance as well as magnitude of differential splicing are derived from an ANOVA based on a mixed linear model.

We generated an exon array data set from clinical samples of NSCLC. Our NSCLC data set contains matched pairs of the AdCa and SCC subtype. Data quality assurance indicated no outlier samples or arrays (additional file 5). In order to identify events of differential splicing we developed a workflow that essentially consists of three components (Figure 1): (1) filtering of probe sets whose signals are not significantly above background signal, (2) re-definition of probe sets according to most up-to-date transcript annotations from public databases, and (3) statistical evaluation using a MLM ANOVA and SI. We have investigated these three components in comparison to standard approaches and outline their particular contributions to a reliable result below.

### Background filtering reduces the number of false positive results

**Figure 2:**
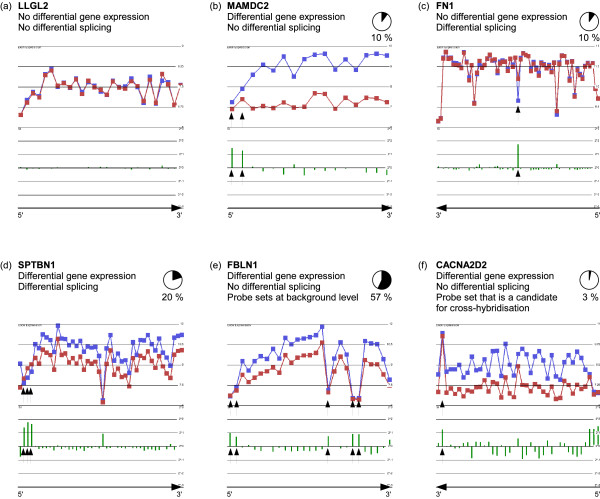
**Expression profiles resulting from the exon array can be classified into one of six classes (representative gene profiles are shown)**. Classification of the top 100 genes generated using the *standard workflow*. Red graph: exon expression in NSCLC; blue graph: exon expression in normal adjacent tissue; green bars: Splicing index per exon (logarithmic scale). **(a) **A gene that is neither affected by differential gene expression nor by differential splicing (none of the top 100 genes falls into this category). **(b) **False-positive result of a differentially expressed gene caused by exons with very low expression (arrows) outside the linear detection range. **(c) **Differential splicing (arrow) in the absence of differential gene expression. **(d) **Differential splicing (arrows) overlaid by differential gene expression. **(e) **Exons showing a constantly low expression value (arrow) that presumably measure the background signal leading to false-positives. **(f) **Exons showing a constantly high expression value (arrow) that presumably measure multiple targets leading to false-positives.

We utilised the generally accepted analysis of variance (ANOVA) method in order to identify gene loci affected by differential splicing. A false discovery rate (FDR) of 0.05 corresponds to an ANOVA p value of 0.018 in the NSCLC data set. According to this analysis, 5340 candidate genes are affected by alternative splicing. Of the genes showing a p value close to zero (*p <*1.4 · 10^-45^), we manually inspected the top 100 list with the most extreme SI, and assigned them to one of six classes according to their expression profile (Figure 2). Although this classification has not been verified and may contain some errors, it will help us to detect key features of an analysis based on ANOVA and SI alone. Representative gene profiles are shown in Figure 2. It became evident that only 30% of all gene loci in the top 100 list are true positives (Figure 2c and 2d). All of the other candidates appear to be false positives (Figure 2b, e, and 2f).

In particular, more than half of all gene loci in the top 100 list exhibited probe sets with a low expression value in both pathology groups (Figure 2e). We assume that these probe sets are *absent *in both pathology groups, i.e. the corresponding exon is expressed neither in tumour nor in NAT. These probe sets will only measure the background signal in the respective sample group and thus are non-informative. Still, their expression value affects the statistical analysis: the FC of absent probe sets does not follow the gene level FC. The statistical ANOVA method scores genes containing such background level probe sets with a low p value which leads to the high rate of 57% false positives. Therefore, we introduced a data set-specific *background filter *that identifies and removes probe sets that are absent in both sample groups before starting any statistical analysis (see Material and Methods).

After applying our background filter, we repeated the ANOVA analysis for the identification of candidates differentially spliced between NSCLC and NAT (FDR := 0.05, *p *= 0.02). This time, we identified 3414 candidate genes. We compared this result set with the result set obtained from the analysis without background filter (5340 candidate genes). There is an overlap of 2505 genes between both sets, i.e. most genes were found with both analysis methods. First, we inspected the set of genes that were removed in the analysis with background filter. Then, we inspected candidate genes that were found only in the analysis with background filter.

In total 2835 genes were removed by the background filter. Of these, 1965 genes were completely removed since less than five of their probe sets were found to be present. Of the remaining 870 genes, in total 4388 absent probe sets were removed by the background filter (31% of all core probe sets of these genes). As a consequence, these genes are no longer identified by the ANOVA method as being differentially spliced. In summary, the background filter removes 53% of all candidate genes from the result list.

However, also 689 additional genes were found to be differentially spliced only after applying the ANOVA analysis with background filter. We hypothesise that the majority are statistically borderline such that their inclusion is due to the effect of setting a cut-off value (here: FDR := 0.05). To test this hypothesis, we analysed the AS ANOVA p values of genes removed by the background filter and genes that were only found with the background filter (in total 1559 genes). p values were taken from the ANOVA analysis both without and with the background filter. For each gene individually, the difference of both log-transformed p values was calculated (Δ log *p*). Under the assumption that our background filter has no effect and all differences are merely an effect of setting a cut-off value, all Δlog *p *values should be small and evenly distributed. We observe a significant skewness in the Δlog *p *value distribution (skewness of -1.77, D'Agostino skewness test *p <*2.2 · 10^-16^). This means that only genes removed by the background filter show highly different p values. Apparently, the background filter has the expected effect and other changes in the result list can be attributed to setting a cut-off value.

### Inappropriate use of annotation file leads to a high false negative rate

Affymetrix provides *meta probe set *(MPS) files that contain relationships between probe sets and genes. MPS files are commonly used (e.g. several implementations in commercial analysis software) to define the set of probe sets constituting a gene, but this is not intended by Affymetrix and leads to a loss of many candidates for differential splicing. We compared this file with supposedly redundant information from more comprehensive chip design annotation files. It became evident that a gene does not contain all available core level probe sets in the MPS file. Affymetrix provides these annotation files for the analysis of differential gene expression and reports that probe sets less suitable for this purpose were left out. We found that by inappropriate use of the annotation file in total 220 differentially spliced genes were lost as false negatives. Our results suggest that by using the original MPS files for the analysis of differential splicing, the false negative rate is unnecessarily increased.

### Further analysis improvement by chip re-annotation with most up-to-date transcript data

**Figure 3:**
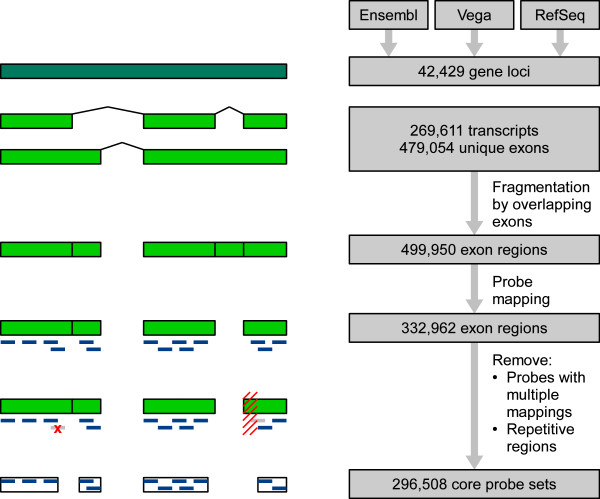
**Workflow for the creation of a new chip definition (definition of probe sets)**. Green boxes: Exons and disjunct exon regions collected from Ensembl 56.37a and RefSeq release 38. Blue: Probes of the exon array. Red: Dropping of probes caused by non-unique mapping of probe sequences or repetitive regions.

As outlined above, it is important to base any analysis of differential splicing upon a proper definition of probe sets and gene loci. In contrast to the identification of novel splice variants, our focus is on finding AS events that are well-annotated by transcript variants in public databases and that are differentially expressed in NSCLC versus NAT. It is intuitive to make use of the best-available transcript annotations. Thus, we created a new chip definition based on recent transcript data (Figure 3).

In order to assess the improvements by the new chip definition, we investigated whether events of differential splicing can be reliably identified. We chose a straightforward approach of manually inspecting gene expression profiles that were generated on the basis of the original or the modified chip definition. The impact on the detection of differential splicing can be most easily assessed with genes where an AS pattern is not overlaid by a differential gene expression pattern. Therefore, we focused on genes that are only affected by differential splicing but that do not exhibit differential expression on the gene level (e.g. Figure 2c). A list of genes was created based on the difference in the absolute SI value: the top end shows results that were gained and the bottom end shows results that were lost by the new chip definition. Expression profiles of the top 10 and the bottom 10 candidates were inspected manually.

It became apparent that almost all of the candidates only identified with the new chip definitions show differential splicing for exons not covered by probe sets in the Affymetrix chip definition. These exons were not annotated as well-known exons before, but are meanwhile supported by new transcript annotations. In most cases, the new probe set contains at least two probes, in one case even nine probes. Thus, additional information on transcript annotations also leads to additional identification of differential splicing events. The probe set of another gene was found to be present in both chip definitions. Only with the new chip definition does this probe set indicate differential splicing. Closer investigation reveals that this probe set contains four probes in the Affymetrix chip annotation. Three of these probes map to multiple locations on the genome and thus were left out in the new chip definition. The new probe set contains only a single, but more reliable probe that now indicates differential splicing. On the top 10 list, we found one case where five genes were combined into a single transcript cluster according to the new chip definition. A single RefSeq transcript annotation spans over five gene loci and thus causes their combination. Differences in the expression levels of the five genes leads to extreme SI values. This example demonstrates that an exon array analysis is sensitive to the definition of transcript clusters. Errors in transcript annotations can lead to artefacts and the example here could represent a false positive result.

For candidate genes that are no longer identified with the new chip definition, we found that in most cases adjacent probe sets were combined to form a single new probe set. The new probe set measures an averaged signal which leads to a reduced SI or no evidence for differential splicing. In two cases, the evidence for differential splicing based on the Affymetrix chip annotation was provided by probe sets containing a single probe. In three cases, probe sets of the Affymetrix chip definition were completely removed. These probe sets contain either a single probe or overlapping probes and furthermore, almost all probes map to repeat masked regions and thus were left out in the new chip definition. Together, combination of probe sets and removal of less reliable probes leads to a new chip definition that is best suited to detect differential splicing of known exons at the expense of the ability to detect novel exons. Our improved workflow now consists of a background filter and a new chip definition which is based on up-to-date transcript annotations. At the same time, the number of artefacts due to cross-hybridisation of probes is reduced.

### Application of the new workflow to reliably identify alternative splicing events

From a previous laboratory validation approach using candidates from the original workflow (Affymetrix chip definition, MLM ANOVA p value) we achieved only a low validation rate. In order to reliably identify genes that are differentially spliced in NSCLC versus NAT, we utilised our *enhanced workflow *(background filter, summarisation using the new chip definition, MLM ANOVA). Based on all available data, differential splicing candidates were selected by an *informed decision-making *approach in order to generate a result list for laboratory validation. In particular, we inspected known transcript variants.

Starting with our new chip definition, we omitted all genes containing fewer than five probe sets (leaving 268132 probe sets that correspond to 16171 genes). After applying the background filter, we generated a primary result list based on the AS ANOVA p value (FDR := 0.05, *p *= 0.02). This generated a *primary result list *of 3096 genes (additional file 6).

**Figure 4:**
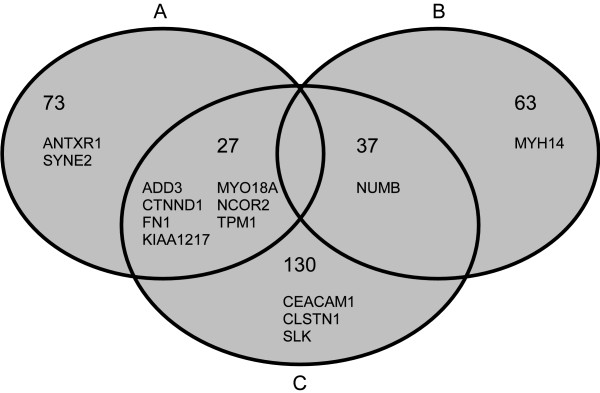
**Venn diagram of the final result lists of genes that are differentially spliced in NSCLC compared to NAT**. From the primary result list of the enhanced workflow (see Figure 1) sub-lists were generated in order to inspect gene expression profiles manually. Genes shown were selected for validation using independent laboratory methods. Sub-list A: Genes that showed evidence for differential splicing and that were not affected by differential gene expression (top 100). Sub-list B: Genes that showed evidence for differential splicing and that were affected by differential gene expression (top 100). Sub-list C: Genes with exons that are known to be involved in alternative splicing and where these exons showed a high splicing index (*SI *≤ 1.4^-1 ^or *SI *≥ 1.4).

Because this list is too large for manual inspection, sub-lists were generated based on additional criteria. *Sub-list A *focuses on genes that are differentially spliced but that are not affected by differential gene expression (see category in Figure 2c). Here, we filtered by pathology group *p >*0.05 and took the top 100 genes with most extreme SI (additional file 7). As we know that most of the alternatively spliced genes are also affected by differential gene expression (see category in Figure 2d), we generated *sub-list B *by filtering pathology group *p *≤ 0.05. Only genes showing high homogeneity in all tumour and all NAT samples (i.e. no dependency on the patient, patient *p >*0.2) were included in this sub-list. Furthermore, we focused on genes where the gene expression FC was not higher than the SI. We took the top 100 genes with most extreme SI (additional file 8). Thirdly, we generated *sub-list C *by focusing on probe sets that are known from transcript annotations (UCSC genome browser, database hg19, table *knownAlt*) to be affected by AS. Genes were included in this sub-list if these specific probe sets had a *SI *≤ 1.4^-1 ^or *SI *≥ 1.4 (in total 194 genes; additional file 9). All expression profiles of genes in any of these sub-lists (Venn diagram Figure 4; additional file 10) were inspected manually in order to formulate a hypothesis on the mode of AS (e.g. cassette exon). Based on all available information, 14 promising candidates were selected for validation by independent methods in the laboratory.

### Laboratory validation of exon array results

Of the 14 candidate genes selected for laboratory validation, Xi et al. already validated differential splicing of *CEACAM1 *in AdCa of NSCLC [34]. For the remaining 13 candidate genes, we hypothesise 10 cassette exon events, two events of an alternative transcription start site, one event of intron retention, one event of an alternative 5'-splice site, and two events of mutually exclusive exons (in total 16 events of differential splicing, see Table 1).

**Table 1:** Validation results of candidate genes.

		*Laboratory results*				
			
Gene	Alt. splicing hypothesis	RT-PCR	Sequencing	qRT-PCR AdCa	qRT-PCR SCC	Status
ADD3	Cassette exon	4/6	+	+	+	validated
ANTXR1	Alt. transcription start site	N/A	N/A	-	-	devalidated
CLSTN1	Cassette exon	4/6	+	+	-	validated
CTNND1	Cassette exon (5')	-	N/A	+	+	devalidated
	Cassette exon (3')	2/6	+	-	-	devalidated
FN1	Cassette exon	5/5	+	+	+	validated
KIAA1217	Alt. transcription start site	N/A	N/A	+	+	validated
	Intron retention	2/6	+	-	-	devalidated
MYH14	Cassette exon	-	+	-	-	devalidated
MYO18A	Cassette exon	4/6	+	+	-	validated
NCOR2	Alternative 5'-splice site	4/6	+	+	-	validated
NUMB	Cassette exon	5/6	+	+	+	validated
SLK	Cassette exon	5/6	+	+	+	validated
SYNE2	Cassette exon	6/6	+	+	-	validated
TPM1	Mutually exclusive exon (5')	N/A	+	+	+	validated
	Mutually exclusive exon (3')	N/A	+	+	+	validated

A hypothesis of the mode of alternative splicing was formulated based on exon array expression profile and available transcript annotations. Events with alternative start of transcription were also considered, although they are not alternative splicing events by definition. RT-PCR was conducted using six samples; shown here is the number of samples that show an alternative splicing pattern that is in concordance with the exon array results. Exon-exon junctions in RT-PCR products were examined by sequencing. qRT-PCR was performed using transcript variant specific primer pairs for both AdCa and SCC. qRT-PCR results confirmed the exon array results (+) if they showed a significant (paired t-test *p *≤ 0.05) or high (*SI *≥ 2.0 or *SI *≤ 2.0^-1^) difference of transcript variant expression that was in agreement with the exon array results. Differential splicing of a gene was considered as validated if at least one of the qRT-PCR results was positive. If available, RT-PCR had to confirm the exon array results in the majority of samples and also sequencing results had to be positive. Detailed result see additional file 11. N/A indicates that the experiment was not performed.

Firstly, we tried to validate all of the cassette exon events, the intron retention event, and the alternative 5'-splice site event using RT-PCR in AdCa samples. In most cases, we identified two distinct PCR products, one originating from the short and the other originating from the long transcript variant of the respective gene (see additional file 11). Both the product size as well as the estimated abundance in tumour versus NAT were in concordance with the exon array results and with the AS event hypothesised by us. However, in two events (*CTNND1-CE-3'*, intron retention event of *KIAA1217*) only two of six patients exhibited the expected result. In two cassette exon events (*CTNND1-CE-5'*, *MYH14*) merely a single PCR product was found. Together, this leads to eight positive and four negative results. In all cases, sequencing of representative PCR products confirmed the expected exon-exon junction sequence according to our AS hypothesis.

Secondly, we tried to quantify the expression of different transcript variants with qRT-PCR. For all of the 16 differential splicing events, transcript variant specific primers were designed. The SI was calculated separately for AdCa and for SCC (based on the qRT-PCR results; see additional file 11). Four events of differential splicing could not be confirmed in AdCa (*ANTXR1*, *CTNND1-CE-3'*, intron retention event in *KIAA1217*, *MYH14*). The remaining events showed a difference of transcript variant expression that is in agreement with the exon array results. High difference (*SI *≥ 2.0 or *SI *≤ 2.0^-1^) was found for ten events (*ADD3*, *CLSTN1*, *CTNND1-CE-5'*, *FN1*, alternative transcription start site in *KIAA1217*, *MYO18A*, *NCOR2*, *NUMB*, *SYNE2*, *TPM1-MX-5'*) and of these, the results of six events achieved significance (*ADD3*, *FN1*, alternative transcription start site in *KIAA1217*, *NCOR2*, *NUMB*, *TPM1-MX-5'*). A smaller yet significant difference was found in two events (*SLK*, *TPM1-MX-3'*). In total, 12 events of differential splicing were confirmed in AdCa. In SCC, eight events exhibited differential splicing that is in agreement with the exon array results (*ADD3*, *CTNND1-CE-5'*, *FN1*, alternative transcription start site in *KIAA1217*, *NUMB*, *SLK*, *TPM1-MX-5'*, *TPM1-MX-3'*). Although the difference in transcript variant expression was high in all cases (*SI *≥ 2.0 or *SI *≤ 2.0^-1^) the significance level was not reached. Three results are borderline (*ANTXR1*, *CLSTN1*, and *MYO18A*) as there was evidence for differential splicing, but neither with significance nor with high magnitude. qRT-PCR results of *NCOR2 *were inverse compared to exon array results (details see additional file 12). Four events of differential splicing were not confirmed with qRT-PCR in SCC (*CTNND1-CE-3'*, intron retention event in *KIAA1217*, *MYH14*, *SYNE2*).

We consider exon array results to be validated if both RT-PCR and qRT-PCR results are in agreement with our hypothesis of AS and with the exon array results (details see Materials and Methods). Based on our validation criteria, 11 events were validated successfully whereas five events were devalidated (see Table 1). Of the latter, two events (*CTNND1-CE-3'*, intron retention event of *KIAA1217*) showed evidence for differential splicing in some patients but failed to meet our relatively strict criteria. Likewise, differential splicing of one cassette exon event of *CTNND1 *(*CTNND1-CE-5'*) was confirmed with qRT-PCR but not with RT-PCR and thus did not meet the validation criteria.

### Expression analysis of FOX splicing factors in NSCLC

Interestingly, many of our validated CE events were previously described as cancer-specific splice variants in breast and ovarian cancer [55]. It was shown that these and AS events of other genes depend on the splicing factor FOX2, which in cancer is either down-regulated or expressed as an inactive splice variant. We tried to elucidate whether FOX2 regulates cancer-specific AS also in other types of cancer such as NSCLC.

**Figure 5:**
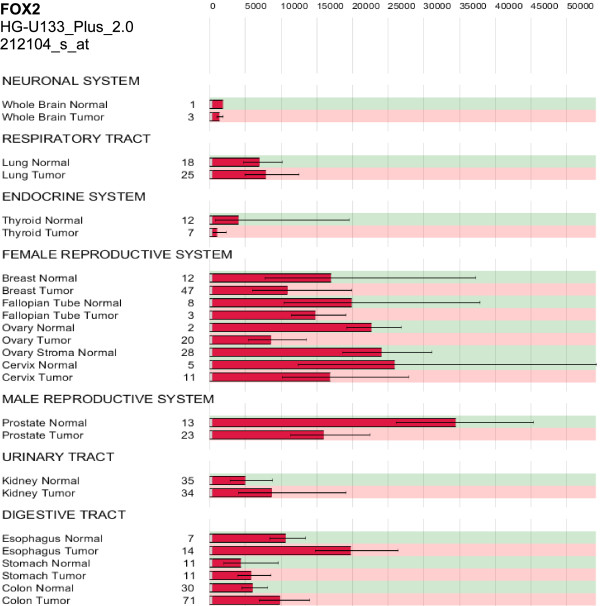
**Expression of FOX2 in twelve different types of cancer and corresponding normal tissue**. Geometric mean signal intensities of probe set 212104_s_at (Affymetrix expression array HG-U133_Plus_2.0) which measures gene expression of FOX2 (*RBM9*). FOX2 was down-regulated in tumour versus normal tissue in strongly hormone-sensitive organs. The numbers of samples per group are shown. Error bars represent one standard deviation as calculated from log-transformed intensities.

First, we analysed expression of the splicing factors FOX1 and FOX2 using a collection of in total 1015 gene expression microarrays. This database consists of twelve types of cancer and corresponding normal tissue as well as 50 other healthy body tissues. FOX1 was only expressed in brain, heart muscle, and skeletal muscle, but neither in cancer nor in other healthy tissues (additional file 13). RT-PCR and qRT-PCR results confirmed no FOX1 expression in NSCLC and NAT (data not shown). FOX2 was down-regulated in tumours of endocrine origin (thyroid, breast, fallopian tube, ovary, cervix, and prostate) but up-regulated in kidney, oesophagus, stomach, and colon cancer. In brain and lung cancer, the FOX2 expression did not change compared to normal tissue (Figure 5, additional file 14).

**Figure 6:**
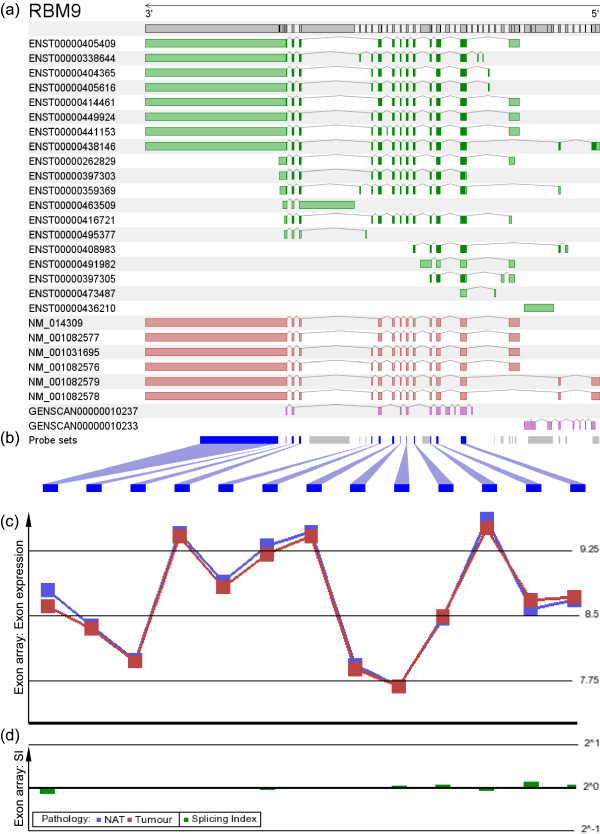
**Details of the exon array results for FOX2 (*RBM9*)**. **(a) **Exon structure and known transcript variants of FOX2 (introns not to scale; green: Ensembl transcripts; red: RefSeq entries; purple: Genscan predictions). **(b) **Position of probe sets in the new exon array chip definition (grey: absent probe sets; blue: present probe sets). **(c) **Exon expression in the NSCLC data set showed no difference between tumour and normal adjacent tissue (NAT) (red graph: exon expression in NSCLC; blue graph: exon expression in NAT). **(d) **Splicing indices for exons in the NSCLC data set (logarithmic scale).

To confirm the expression levels and to investigate AS of FOX genes in lung cancer, we examined the respective exon expression profiles in our NSCLC exon array data set. All probe sets corresponding to exons of FOX1 were found to be absent (data not shown). None of the FOX2 probe sets showed any indication of differential expression or differential splicing (Figure 6). The exon array results confirmed the data from our gene expression microarray database.

**Figure 7:**
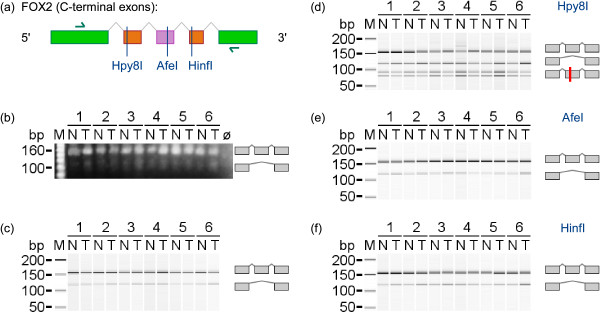
**Alternative splicing of FOX2 affecting exons in the C-terminal region**. **(a) **Exon structure (green: constitutive exons; orange: annotated cassette exons; purple: putative cassette exon predicted by orthology), location of RT-PCR primers (green arrows), and unique restriction enzyme cleavage sites (exon and primer sizes are shown to scale, introns not to scale). **(b) **Verification of RT-PCR product sizes generated from paired samples of adenocarcinoma of NSCLC and NAT of six patients (ø: no template control). **(c) **RT-PCR product sizes analysed using the Bioanalyzer. **(d) **Cleavage by Hpy8I indicated presence of the first cassette exon. **(e) **No cleavage by AfeI indicated absence of the second cassette exon. **(f) **No cleavage by HinfI indicated absence of the third cassette exon.

Although no evidence for a change between NSCLC and NAT was obtained, we wanted to prevent a false negative result and analysed FOX2 expression using PCR based methods. For the analysis of AS of FOX2, we focused on exons in the C-terminal region whose skipping was shown to generate an inactive form of the splicing factor [56]. We noted that two cassette exons are annotated in this region. In a previous study of the mouse orthologue of FOX2, yet another cassette exon designated as M43 was described [57]. An orthologous open reading frame in the intron sequence of human FOX2 is also present and we included it as a putative exon in our analysis. Primers for RT-PCR assays were placed in the flanking constitutive exons. The experimental results showed that two transcript variants were present both in NSCLC as well as in normal lung tissue (Figure 7). The smaller RT-PCR product resembled an amplicon lacking all cassette exons whereas the longer product allowed the inclusion of only one of the three cassette exons. All cassette exons were of similar size, hence exon-specific restriction enzymes were used in order to identify the exons involved by cleavage. DNA fragments after restriction digestion indicated that only the first of the three cassette exons was expressed. This was the same exon that had previously been reported as being skipped in breast cancer [55].

**Figure 8:**
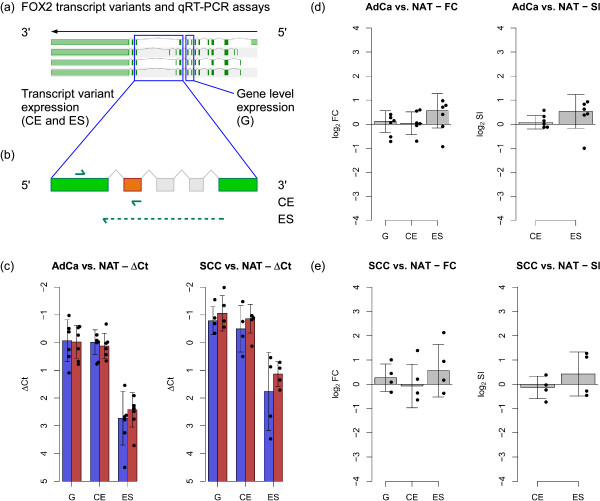
**Quantification of FOX2 transcript variant expression**. **(a) **Four of the annotated transcript variants of FOX2 (for complete set, see Figure 6a) and location of qRT-PCR assays that measure gene level expression and transcript variant expression, respectively. **(b) **Location of primers for transcript variant specific qRT-PCR assays in the C-terminal region of FOX2 (green: constitutive exons; orange: cassette exon; grey: cassette exons not expressed; green arrows: primers; dotted arrow: junction primer). **(c) **Quantification of gene expression (G) and transcript variant expression (CE: cassette exon; ES: exon skipping) in adenocarcinoma and squamous cell carcinoma of NSCLC (red bars), respectively, compared to NAT (blue bars). Bars indicate median Δ*Ct *values, dots represent values for individual samples (AdCa: *n *= 6; SCC: *n *= 4), error bars indicate one standard deviation. **(d) **Fold-change (FC) of over-expression in adenocarcinoma of NSCLC versus NAT and splicing index (SI). Median values based on six sample pairs are shown (values for each patient shown as dots), significance was determined using a paired t-test. **(e) **Fold-change of over-expression in squamous cell carcinoma of NSCLC versus NAT and splicing index. Median values based on four sample pairs are shown.

The overall FOX2 expression levels and expression levels of each of the two transcript variants were quantified using specific qRT-PCR assays. It became evident that the longer FOX2 transcript variant, which includes the first cassette exon, was the dominating form and was expressed up to six-fold higher than the smaller transcript variant. But neither the FOX2 gene expression nor expression of any transcript variant differed significantly between NSCLC and NAT (Figure 8).

### Quantification of *ADD3 *transcript variants in NSCLC

**Figure 9:**
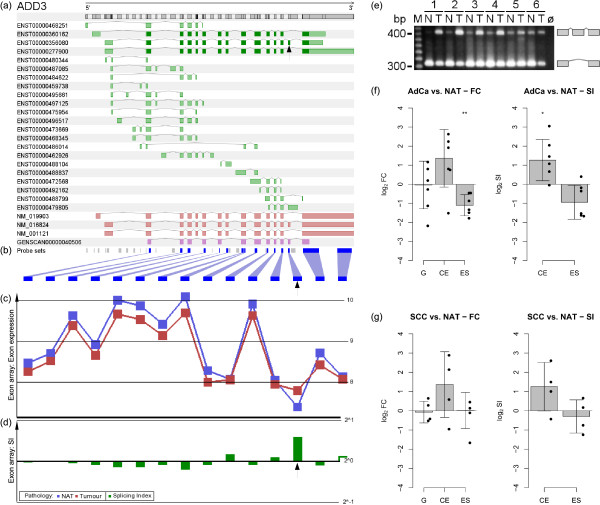
**Details of the exon array results and the laboratory validation results for *ADD3***. **(a) **Exon structure and known transcript variants of *ADD3 *(introns not to scale; green: Ensembl transcripts; red: RefSeq entries; purple: Genscan predictions). **(b) **Position of probe sets in the new exon array chip definition (grey: absent probe sets; blue: present probe sets). **(c) **Exon expression in the NSCLC data set suggests higher inclusion of a cassette exon (arrow) in tumour compared to normal adjacent tissue (NAT) (red graph: exon expression in NSCLC; blue graph: exon expression in NAT). **(d) **Splicing indices for exons in the NSCLC data set (logarithmic scale). **(e) **Verification of RT-PCR product sizes generated from paired samples of adenocarcinoma of NSCLC and NAT of six patients (ø: no template control). In tumour, exon inclusion was observed in all cases analysed. Sequencing of representative products confirmed the expected exon-exon junctions (data not shown). **(f) **Quantification of gene expression (G) and transcript variant expression (CE: cassette exon; ES: exon skipping) in adenocarcinoma of NSCLC compared to NAT as measured by qRT-PCR. Median values based on six sample pairs are shown (values for each patient shown as dots), error bars indicate one standard deviation, significance was determined using a paired t-test. FC: Fold-change of over-expression in adenocarcinoma of NSCLC versus NAT. SI: Splicing index. **(g) **Quantification of gene expression and transcript variant expression in squamous cell carcinoma of NSCLC compared to NAT as measured by qRT-PCR. Median values based on four sample pairs are shown.

One of the successfully validated genes that showed a significant validation result is now described in more detail: *ADD3*. *ADD3 *contains at least 16 exons of which exon number 15 (ENSE00000986819) is a known cassette exon of 96 bp. In Ensembl, 22 transcript variants of *ADD3 *are annotated (Figure 9a). The exon array probe set that maps to the cassette exon exhibited a high SI in the NSCLC exon array data set (Figure 9b-d). This indicates that the cassette exon is preferably included in cancer, but skipped in normal tissue. This differential splicing pattern was detected in 13 of 18 patients (72%). Four patients showed an inverse pattern and one patient did not exhibit any indication of differential splicing. There was no correlation between these differences and factors such as subtype or staging. RT-PCR of AdCa samples confirmed the differential splicing pattern measured using the exon array in four cases (Figure 9e samples 1 - 4). Furthermore, in two patients (samples 5 and 6) AS clearly took place, but no difference was observed between tumour and NAT. These were the same patients that showed an inverse result or no differential splicing on the exon array. Each transcript variant was quantified with qRT-PCR using specific primer pairs. Moreover, the overall gene expression of *ADD3 *was measured using a primer pair targeting a constitutive gene region. qRT-PCR results (Figure 9f-g) confirmed that the overall amount of *ADD3 *did not change between tumour and NAT; thus, FC and SI values were similar. Both in AdCa as well as in SCC a switch from exon skipping to exon inclusion was observed such that the cassette exon was expressed more than 2-fold higher in tumour compared to NAT. Likewise, the exon skipping transcript variant was expressed about 2-fold lower in tumour versus NAT; however, the latter difference was only observed in AdCa, not in SCC. Based on these results, we postulate that *ADD3 *lacking the cassette exon is expressed in normal lung tissue. In NSCLC, a switch occurs to exon inclusion while the overall gene expression remains unchanged.

### Alternative splicing patterns that correlate with the NSCLC sub-type

The laboratory validation results demonstrated that we could reliably identify genes that are differentially spliced from an exon array data set using the improved workflow. Finally, we investigated whether there are genes whose splicing pattern shows a clear difference that correlates with the NSCLC subtype, i.e. AdCa and SCC. Our data set contains a balanced set of AdCa and SCC samples. In the ANOVA model, the factor subtype (levels: AdCa, SCC, and NAT) was used instead of factor pathology (levels: tumour and NAT). In total, 4285 genes showed evidence of differential splicing in at least one subtype (FDR := 0.05, *p *= 0.026; additional file 15). For all samples and subtypes, we calculated separate SI values. A high difference of SI values between AdCa and SCC is an indication of a subtype-specific pattern of AS. There were 16 genes that exhibited a subtype-specific AS pattern (Δ *log*_2_*SI *≥ 0.5 or Δ *log*_2_*SI *≤ -0.5). In each gene, we analysed the probe set with the highest difference in SI values between AdCa versus NAT compared to SCC versus NAT. With regard to these probe sets, we found that three genes exhibited a more pronounced differential splicing pattern in AdCa (*GTPBP10*, *DUOX1*, and *KIAA1217*), whereas 13 genes had a more pronounced pattern in SCC (e.g. *CD44*, *FN1*). In the case of *KIAA1217*, the respective probe set covered the exon that had been quantified using qRT-PCR. In agreement with the exon array expression values, also qRT-PCR results showed an increased expression of the short transcript variant that was more pronounced in AdCa versus NAT compared to SCC versus NAT.

## Discussion

Despite novel targeted therapies that are available or in development, treatment of NSCLC remains a major unmet clinical need. Based on the analysis of individual genes, it is known that AS occurs and plays an important role in cancer. In this study, we present and analyse a data set of matched pairs of clinical samples of NSCLC measured with the exon array technology, with which one can investigate AS genome-wide. We elucidate the relatively unexplored field of AS in NSCLC and analyse selected genes in more detail.

### Data set

Clinical samples show high variability. This can be attributed to patient-to-patient variability and in the case of cancer, to differences in tumour content and especially to considerable differences between individual tumours. We only used matched pairs of NSCLC and NAT in order to level out patient-to-patient variations. Tumour content was high in all NSCLC samples and a sub-set of the samples was enriched by macrodissection. The data set is balanced such that both major sub-types of NSCLC (AdCa and SCC) and staging are present in similar proportions. Although detailed sample annotations are available, finding AS patterns specific to each factor remains a question of power. With 18 sample pairs our NSCLC data set has the power to discriminate between tumour and NAT on a level similar to other cancer data sets with matched samples. While Xi et al. focused on the AdCa sub-type of NSCLC [34], our data set also contains the SCC sub-type. This allows identification of AS patterns common to both AdCa and SCC. In addition, one can find sub-type specific AS patterns.

### Workflow improvements

It is equally important to use an analysis workflow with a high detection rate and a justifiable false positive rate. We found that the *standard workflow *leads to a low true positive rate of 30% caused primarily by artefacts. We introduced an enhanced analysis workflow consisting of (1) filtering of probe sets that are absent in both of the sample groups, (2) an updated chip annotation, and (3) a statistical analysis based on a mixed linear model (MLM) ANOVA and SI.

A modified version of the model-based algorithm MAT was used in order to identify *absent probe sets*. Rather stringent cut-off values were set with regard to the MAT algorithm p value as well as homogeneity in one sample group in order to flag a probe set as absent. Thus, genes with low expression and signal intensities close to the background noise might have got lost as false negatives. Nevertheless, these background filter settings resulted in a pronounced decrease of artefacts which we consider to be essential for a reliable result list.

We showed that the analysis can be further improved by an *updated chip annotation *based on recent transcript data. Thus, differential splicing of new exons that were not annotated as well-known at chip design time can now be detected. In contrast to the chip definition created by Affymetrix, we did not include EST data or predicted exons in order to avoid excessive clustering of exons. Probes that map to multiple locations to the genome as well as probes mapping to repeat-masked regions were left out during the reannotation process in order to avoid artefacts caused by cross-hybridisation. We found evidence that this approach leads to probe sets with a higher number of probes and thus a more reliable measurement of exon expression. Different parts of an exon that has a predicted internal splice site can obviously not be discriminated. Our chip definition is best suited to analyse differential splicing of known transcript variants in contrast to the detection of novel transcript variants. It should be noted that the new chip definition can be used in combination with any algorithm of choice and within other analysis workflows than the one described in this study. All files are compatible with the respective Affymetrix file format and no changes in software implementations are necessary.

Thirdly, we introduced an informed decision-making approach for the identification of genes that are differentially spliced. A *MLM ANOVA *yielded the significance of AS, differential gene expression, and information about heterogeneity between the patients. In addition, we estimated the gene level expression value directly from the MLM by calculating a contrast. We assume that this is a straight yet robust approach for reliably calculating the SI. Potential candidate genes that are affected by differential splicing were identified using all information available including SI, significance, known events of alternative splicing, and transcript annotations. In effect, we used advanced statistical tools to generate a primary result list. Based on all information available, we selected genes from this list and inspected their exon expression profile manually.

The *enhanced workflow *was applied to our NSCLC exon array data set. A validation of the representative candidates using independent laboratory methods led to a 69% confirmation rate of exon array data. In addition, we found genes that are fairly known to be affected by AS in cancer, e.g. *FN1*. We did not adjust our workflow to give better results for well-known cancer genes. That these kind of genes can be found on the result list confirms that enhancements led to a more reliable identification of differential splicing in general.

### Differential splicing in non-small cell lung cancer

In this study, we found that 330 genes are affected by differential splicing in NSCLC compared to NAT. We investigated their expression profiles and moved on to the next step, the formulation of a hypothesis of AS, i.e. definition of the exons involved and the mode of AS (e.g. cassette exon). Of the primary result list, 14 candidate genes were selected and validated by the independent laboratory methods RT-PCR and sequencing. In total, validation results confirmed 11 events of differential splicing in NSCLC. One of these events represents usage of an alternative start of transcription which is not strictly a mode of AS and might be regulated at a different level than splicing. The other events, however, are genuine events of AS. Several of the successfully validated genes have already been shown to be differentially spliced in cancer. Our data confirmed a switch to the onco-foetal EDB antigen in *FN1*. *CLSTN1*, which is a transmembrane and cell adhesion protein, was found to be affected by AS in colon, breast, bladder, and prostate cancer before [32,58]. Dutertre et al. showed that expression of *CLSTN1 *transcript variants can be utilised in order to give a prognosis of metastasis-free survival in breast cancer [58]. We found that exon skipping in *CLSTN1*, which Dutertre et al. demonstrated as being associated with metastasis in breast cancer, occurs in NSCLC. *FN1 *and *CLSTN1 *were also reported in another study as being differentially spliced in breast and ovarian cancer versus normal tissue [55]. Closer investigation of the primers used in the RT-PCR revealed, however, that exons other than the cassette exons that we found being differentially spliced in NSCLC were affected. *CEACAM1 *is a transmembrane protein that is involved in signal transduction. One alternatively spliced cassette exon leads to a short isoform. Xi et al. showed qRT-PCR results indicating that the short isoform of *CEACAM1 *is over-expressed in both AdCa and SCC of NSCLC compared to NAT [34]. In our exon array data set, the signal intensity of the respective probe set was at the background level and hence no conclusion could be made. For another cassette exon in *CEACAM1*, however, our exon array data set indicated a higher skipping rate in NSCLC compared to NAT. Tropomyosin 1 (*TPM1*) contains two pairs of mutually exclusive exons. Recent transcript annotations in Ensembl 56.37a provide evidence that the first pair is not strictly mutually exclusive since both exons were found together in two transcript variants. *TPM1 *is a tumour suppressor that is involved in cytoskeleton remodelling and that has pro-apoptotic properties [59]. With regard to the first exon pair (exon 2 and exon 3), our results provided evidence for a switch from exon 2 to exon 3 in NSCLC. The exon pair 7A/7B was found to be differentially spliced in different kinds of cancer (colon, bladder, kidney, lung, prostate, and uterus [60,27,32]). Our results confirmed that the mutually exclusive exon pair 7A/7B of *TPM1 *is differentially spliced in NSCLC such that there is a switch from exon 7B to exon 7A in tumour. The alternative 5'-splice site in one exon of *NCOR2 *leads to different gene products that are known as SMRT-*α *(long exon) and SMRT-*τ *(short exon) [61]. In this study we demonstrated that in normal lung tissue both transcript variants of *NCOR2 *are equally expressed; however, at least in NSCLC AdCa a strong shift towards the longer transcript variant SMRT-*α *was observed. In *KIAA1217*, usage of an alternative start of transcription leads to a shorter transcript that is in-frame. To our knowledge, cancer-specific usage of an alternative start of transcription in *KIAA1217 *has not been reported before. In the genes *ADD3*, *MYO18A*, *NUMB*, *SLK*, and *SYNE2*, AS events lead to in-frame variations in the protein sequence affecting from 15 to 48 amino acids.

### FOX2 activity is not regulated at the transcript level in NSCLC

Venables et al. found that many cancer-specific AS events in breast and ovarian tumours are caused only by alterations of the splicing factor FOX2 (*RMB9*) [55]. Among other genes, this was shown for *ADD3*, *MYO18A*, *NUMB*, and *SYNE2*. FOX2-dependent AS was demonstrated for *SLK *in a study of human embryonic stem cells [62]. Thus, many of the cassette exon events of which we confirmed cancer-specific AS in NSCLC are known to be regulated by FOX2. This led us to analyse whether FOX splicing factors (FOX1 or FOX2) could possibly explain the changes in splicing patterns between NSCLC and normal lung tissue. FOX1 shows only limited expression in healthy tissue [63,64] and is expressed neither in breast nor in ovarian tissue [55]. Our results demonstrated that FOX1 is not expressed in normal lung tissue or in NSCLC, either. Two distinct mechanisms were identified by which FOX2 modifies the splicing pattern of target genes in cancer [55]. In ovarian cancer, FOX2 expression is significantly down-regulated compared to normal ovarian tissue. FOX2 itself is also affected by AS in breast cancer and a shift towards a functionally inactive splice variant was observed before. Our gene expression microarray data confirmed that the overall FOX2 expression level is down-regulated in ovarian cancer compared to normal ovary. Also other tumours of endocrine origin in our microarray database showed this kind of down-regulation (thyroid, breast, fallopian tube, cervix, and prostate). Expression of FOX2 was, however, not changed between lung cancer and normal lung tissue. Furthermore, other types of cancer (kidney, oesophagus, stomach, and colon cancer) showed an even higher expression of FOX2. Our analysis of splice variants revealed that the functional variant as well as the inactive form of FOX2 is expressed in lung tissue. A change of the splicing pattern between NSCLC and normal lung tissue was not observed. This means that there is no shift to the inactive FOX2 transcript variant in NSCLC in contrast to breast cancer. These findings suggest that FOX2 activity is not regulated at the transcript level in NSCLC. With the present data, regulation of FOX2 activity at the protein level cannot be excluded. Another explanation is that FOX splicing factors, unlike in other types of cancer, are not responsible for the cancer-specific splicing patterns in NSCLC. FOX2-dependent regulation of AS might be linked to steroid hormone systems that have a significant influence on reproductive system tissues like ovary, cervix, breast, and prostate. This can be explained for instance by the known interaction of FOX2 protein with the oestrogen receptor and transcription factor ER (*ESR1*) [65].

### The long isoform of the cytoskeleton protein ADD3 is cancer-specific

It was also noted by Venables et al. that many of the cancer-associated genes found in their study are functionally related to remodelling of the cytoskeleton and cell movement [55]. Most of the candidate genes of which we confirmed cancer-specific AS in NSCLC are also involved in these processes (*ADD3*, *CLSTN1*, *FN1*, *MYO18A*, *NUMB*, *SYNE2*, and *TPM1*). This is another indication that AS potentially plays a major role in tumour progression, especially in tumour invasion and metastasis. We analysed AS of one gene that is functionally involved in the cytoskeleton in detail.

Gamma-adducin (*ADD3*) is a structural constituent of the spectrin-actin cytoskeleton that contains one known cassette exon. We found that this cassette exon has higher inclusion in NSCLC compared to NAT for the majority of patients. Dutertre et al. found that AS of the same cassette exon is associated with metastasis: they analysed AS in primary tumours generated from four different mouse mammary tumour cell lines with different potential for metastasis. Cassette exon inclusion in the mouse ortholog of *ADD3 *is high only in tumours originating from cell lines with a high potential for metastasis [58]. Interestingly, *ADD3 *was also detected in acute lymphoblastic leukemia as a fusion protein with *NUP98 *[66]. We investigated the gene structure of *ADD3 *in more detail in order to examine potential functional consequences of AS. *ADD3 *encodes the *γ *polypeptide chain of adducin. Adducin occurs as a heterodimer or heterotetramer of *α *adducin together with either *β *adducin or *γ *adducin. The function of adducin oligomers in cells is capping (+)-ends of actin filaments (*f*-actin) and recruitment of spectrin [67]. All adducin monomers have a similar structure: a globular head domain that participates in subunit interaction, a neck domain, and an unstructured tail domain. At the C-terminus, a sequence with homology to myristoylated alanine-rich C kinase substrate (MARCKS) protein can be found that contains the spectrin/actin interaction site as well as calmodulin and protein kinase C (PKC) targets [68]. In *ADD3*, the cassette exon can generate an insert of 32 amino acids in the tail domain that is upstream of the MARCKS-related domain. Protein structure predictions from Ensembl 56.37a provided information that this insert leads to a small coiled coil formation followed by a low complexity region. With the present data, we cannot estimate what effect the amino acid sequence insert generated by AS has on either the oligomerisation of adducin monomers or the binding of other proteins like *f*-actin, spectrin, calmodulin, and PKC.

While our exon array analysis and the validation process focused on finding events of AS that were present in the majority of patients in our study, we also found evidence for considerable heterogeneity, e.g. four of 18 patients showed an inverse AS pattern for *ADD3 *and an inconclusive result for *NCOR2 *in SCC. We gave an outlook as to how additional factors like tumour subtype can be considered in our workflow. In total, 16 genes showed an AS pattern that is differentially pronounced in AdCa versus SCC. Yet, inclusion of additional factors is a matter of power and hence study design.

## Conclusions

With the NSCLC exon array data set presented here, we identified and successfully validated genes that exhibit differential splicing in NSCLC compared to NAT. These genes are involved in processes like apoptosis, cytoskeleton remodelling, and angiogenesis. This underlines the importance of AS in cancer with regard to key processes of cancer progression. We showed that the activity of FOX2, the splicing factor shown to cause cancer-specific splicing patterns in breast and ovarian cancer, is not altered in NSCLC at the transcript level. Either cancer-specific alternative splicing in NSCLC does not depend on the splicing factor FOX2 or other regulatory mechanisms of FOX2 activity, such as at the protein level, are yet to be discovered. Genes affecting the splicing machinery or genes up-stream in the signal transduction cascade are of special interest in the search for novel targeted therapeutics. With regard to drug research, it is therefore desirable to further elucidate the splicing factor network in NSCLC and other types of cancer.

## Abbreviations

AdCa: Adenocarcinoma; ANOVA: Analysis of variance; AS: Alternative splicing; CE: Cassette exon; ES: Exon skipping; EST: Expressed sequence tag; FC: Fold change; FDR: False discovery rate; IR: Intron retention; MLM: Mixed linear model; MPS: Meta probe set; MX: Mutually exclusive exons; NAT: Normal adjacent tissue; NSCLC: Non-small cell lung cancer; PSR: Probe selection region; qRT-PCR: Quantitative reverse transcription PCR; RT-PCR: Reverse transcription PCR; RIN: RNA integrity number; SCC: Squamous cell carcinoma; SI: Splicing index.

## Competing interests

The authors declare that they have no competing interests.

## Authors' contributions

WL participated in the study design, carried out microarray hybridisation, performed the statistical analysis, developed the analysis workflow, generated the new chip definition, carried out the laboratory validation experiments, performed the expression analysis of FOX genes, and drafted the manuscript. FS participated in the design of the study, the statistical analysis, development of the analysis workflow and helped to draft the manuscript. GL participated in the design of the study and microarray hybridisation. GB helped to develop the analysis workflow and to draft the manuscript. HS participated in the design of the study, the statistical analysis, and the development of the analysis workflow. JG and MH provided clinical sample material, performed the pathological analysis and tumour classification. AS conceived of the study, and participated in its design and coordination and helped to draft the manuscript. All authors read and approved the final manuscript.

## Supplementary Material

Additional file 1 **Table S1: Tissue specimens**. Clinical information for all paired tissue specimens. Click here for file

Additional file 2 **Data files S2: New probe set definition**. ZIP-archive containing the new probe set definition provided as a probe group file (PGF), probe set and transcript cluster annotations in CSV format, and a meta probe set file defining the relationship between transcript cluster and probe sets. All file formats are in accordance with Affymetrix definitions. Click here for file

Additional file 3 **Table S3: Endpoint RT-PCR assays**. Primer pairs for RT-PCR assays and amplicon lengths of the expected transcript variants. Click here for file

Additional file 4 **Table S4: Quantitative RT-PCR assays for detection with SYBR™**. Primer pairs for transcript variant specific qRT-PCR assays together with the expected amplicon length. Click here for file

Additional file 5 **Text S5: Quality assurance of the NSCLC exon array data set**. Quality assurance of the NSCLC exon array data set by principal component analysis and hierarchical clustering. Click here for file

Additional file 6 **Table S6: Analysis of the exon array NSCLC data set, primary result list**. Click here for file

Additional file 7 **Table S7: Analysis of the exon array NSCLC data set, result sub-list A**. Click here for file

Additional file 8 **Table S8: Analysis of the exon array NSCLC data set, result sub-list B**. Click here for file

Additional file 9 **Table S9: Analysis of the exon array NSCLC data set, result sub-list C**. Click here for file

Additional file 10 **Table S10: Analysis of the exon array NSCLC data set, final result list**. The final result list is the union of sub-lists A, B, and C (additional file 7, 8, and 9, respectively). Click here for file

Additional file 11 **Table S11: Validation results of candidate genes**. Click here for file

Additional file 12 **Text S12: Quantification of NCOR2 transcript variants in NSCLC**. Click here for file

Additional file 13 **Figure S13: Expression of FOX1 in twelve different types of cancer and corresponding normal tissue as well as in 50 other healthy tissues**. Geometric mean signal intensities of probe set 1553422_s_at (Affymetrix expression array HG-U133_Plus_2.0) which measures gene expression of *A2BP1 *(FOX1). The number of samples per group is shown (in total, 1015 samples). Error bars represent one standard deviation as calculated from the log-transformed intensities. Click here for file

Additional file 14 **Figure S14: Expression of FOX2 in twelve different types of cancer and corresponding normal tissue as well as in 50 other healthy tissues**. Geometric mean signal intensities of probe set 212104_s_at (Affymetrix expression array HG-U133_Plus_2.0) which measures gene expression of *RBM9 *(FOX2). The number of samples per group is shown (in total, 1015 samples). Error bars represent one standard deviation as calculated from the log-transformed intensities. Click here for file

Additional file 15 **Table S15: Result list differential splicing in correlation to the NSCLC subtype**. Click here for file
