# Supplementary material for: Exon Array Analysis using re-defined probe sets results in reliable identification of alternatively spliced genes in non-small cell lung cancer
Source: BMC Genomics. 2010 Nov 30;11:676. doi: 10.1186/1471-2164-11-676 (PMC3053589; doi:10.1186/1471-2164-11-676)
Supplement: Additional file 5 — Text S5: Quality assurance of the NSCLC exon array data set. Quality assurance of the NSCLC exon array data set by principal component analysis and hierarchical clustering. [file 1471-2164-11-676-S5.PDF]

S5: Quality assurance of the NSCLC exon array data set

Obvious outlier experiments were neither observed during the laboratory process nor by visual inspection of the images of scanned arrays. It can be assumed that all samples share common expression patterns. If any differences are present, correlation with the biology of the sample is likely to be found. One example might be a cluster of all tumours versus a cluster of all NAT samples. Should a single sample show a completely different expression pattern, i.e. it does not cluster with other samples, this can be an indication of a biological outlier, faulty preparation, or major microarray defects. It is accepted practice in microarray quality assurance to use independent global analysis methods in order to detect outliers. In this study, raw probe intensities were adjusted for the background signal and summarised to probe set intensities in pre-processing steps. Before the final normalisation of the probe set intensities, all of these values were globally analysed employing three methods: principal component analysis (PCA), hierarchical clustering, and distribution analysis.

PCA involves a transformation of all intensity values into uncorrelated variables called principal components. The first principal component explains as much variability as possible. Each succeeding principal component accounts for as much of the remaining variability as possible. Only the first, second, and third principal components of each sample were displayed in a 3D-scatter plot (Supplementary fig. S5–1). A clear separation between almost all tumour samples and NAT samples became evident. In one patient (L1784), the NAT sample clustered together with the tumour samples which might indicate that the adjacent tissue has in fact a high tumour content. Two of the tumour samples (L9 and L14) were found at a distance to the remaining tumour samples. The same result was obtained with unsupervised hierarchical clustering (Supplementary fig. S5–2). Again, two clusters reflecting the pathology are formed. Notably, the NAT sample of patient L1784 was found to be very similar to the tumour samples. Other samples show a higher distance to the prominent tumour and NAT clusters, including the tumour samples of patients L9 and L14. Finally, the distributions of the probe set intensities were compared between the samples. No obvious outlier was identified from the box plot (Supplementary fig. S5–3). All samples show almost identical quartiles and whiskers, which represent the 10 %- and the 90 %-quantile, respectively. The spread of the tumour sample of patient L9 was found to be slightly smaller.

PCA and hierarchical clustering were also performed using gene level expression values. To this end, all probe set intensities of a gene were summarised to the gene level intensity. PCA of gene level intensity values again showed a clear separation between tumour and NAT samples with the exception of patient L1784 (Supplementary fig. S5–4). Again, tumour samples of two patients (L9 and L14) were found to be more distant from the other samples. Also unsupervised hierarchical clustering of gene level intensity values reveals clustering by pathology (Supplementary fig. S5–5). The NAT sample of patient L1784 was found in the tumour cluster. The tumour samples of patients L9 and L14 still show a high distance; yet, two other samples (L2 and L6152) show a higher distance. Results of the global analysis using gene level expression reconfirm the results obtained using exon level expression values.

Together, the global analysis methods demonstrated that there is a distinction in the exon and gene expression pattern between tumour versus NAT. As expected from the tumour biology, the tumour samples are less homogenous than normal lung tissue specimens adjacent to the tumour. Compared to the overall sample collective, NAT of patient L1784 could possibly have a high tumour content. It was, however, not classified as an outlier since the statistical hypothesis tests are sufficiently robust to tolerate a single potential outlier. All tumour samples display diversification, with those of patients L9 and L14 showing the highest distance. As this demonstrates the diversity of individual NSCLC tumours, neither sample L9 nor L14 was flagged as an outlier. From the technical point of view, all microarrays appear to be of high quality and were utilised in the analysis of alternative splicing in NSCLC.

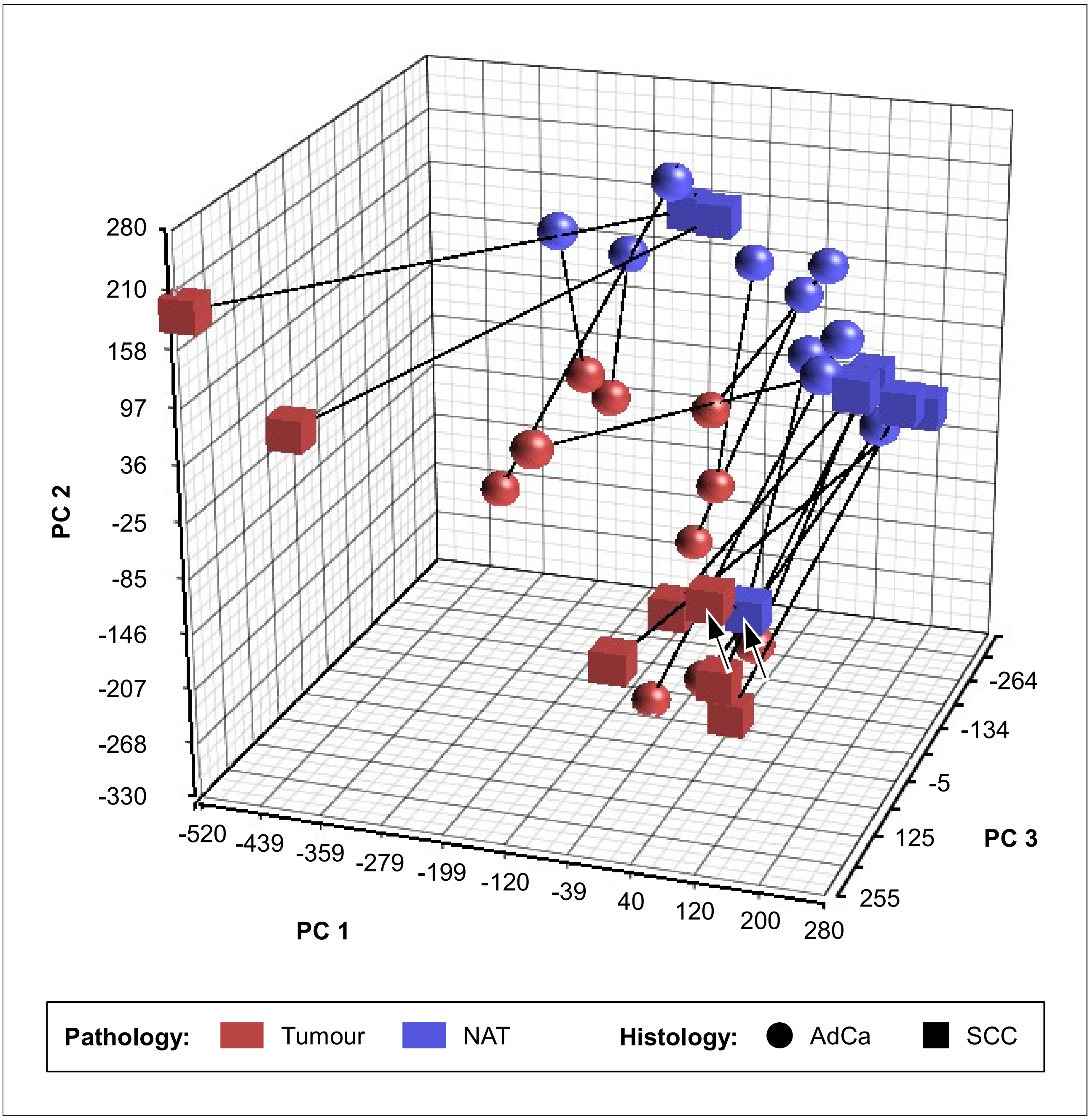

Supplementary figure S5–1: Principal component analysis of probe set level intensities. The first, second, and third principal components (PC) are shown in a 3D-scatter plot. Paired samples of a patient are connected with a line. Patient L1784 is located in the centre (highlighted by arrows). Tumour samples of the patients L9 and L14 are located in the top-front-left octant.

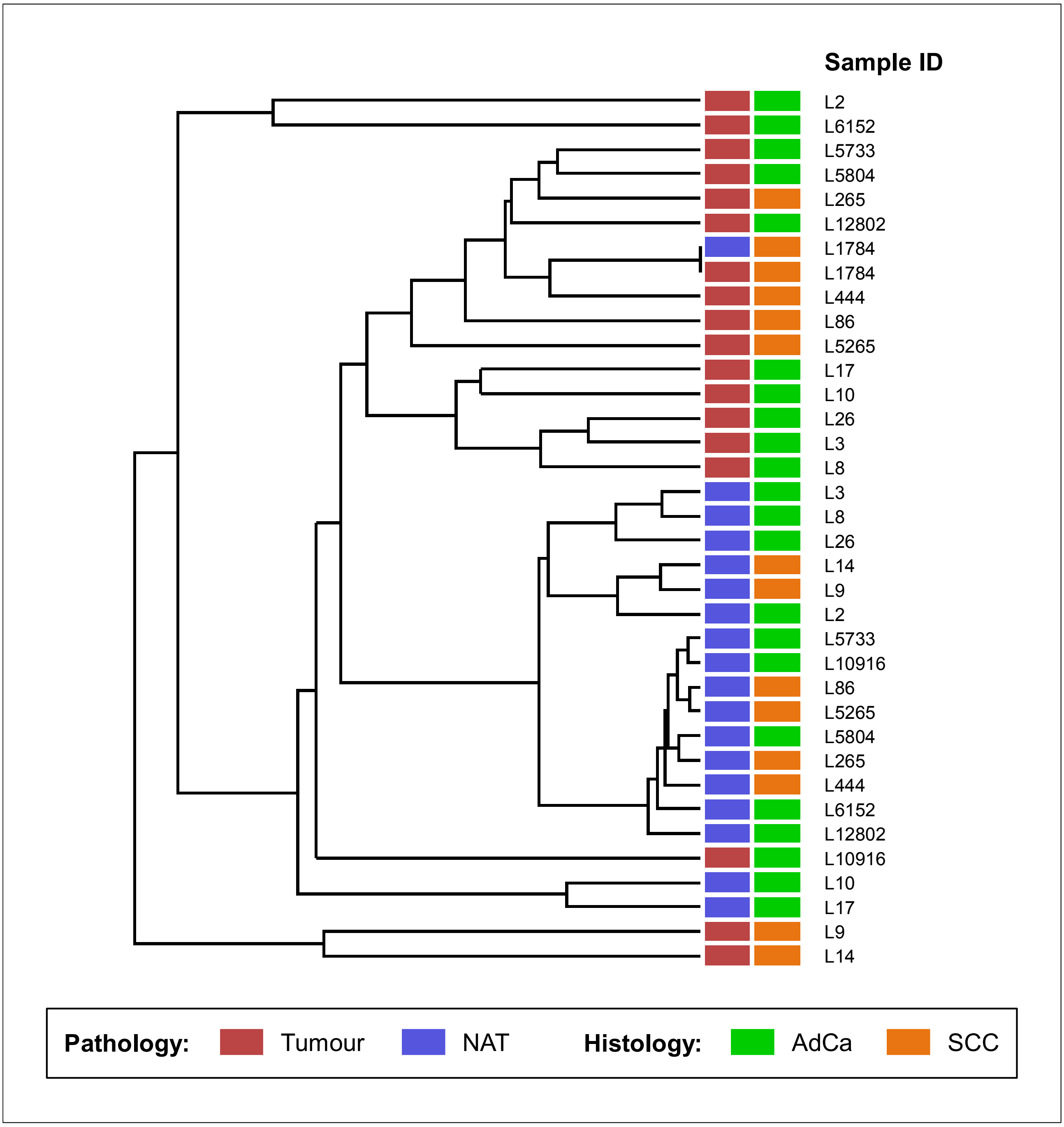

Supplementary figure S5–2: Unsupervised hierarchical clustering of probe set level intensities. Pearson's dissimilarity metric, average linkage.

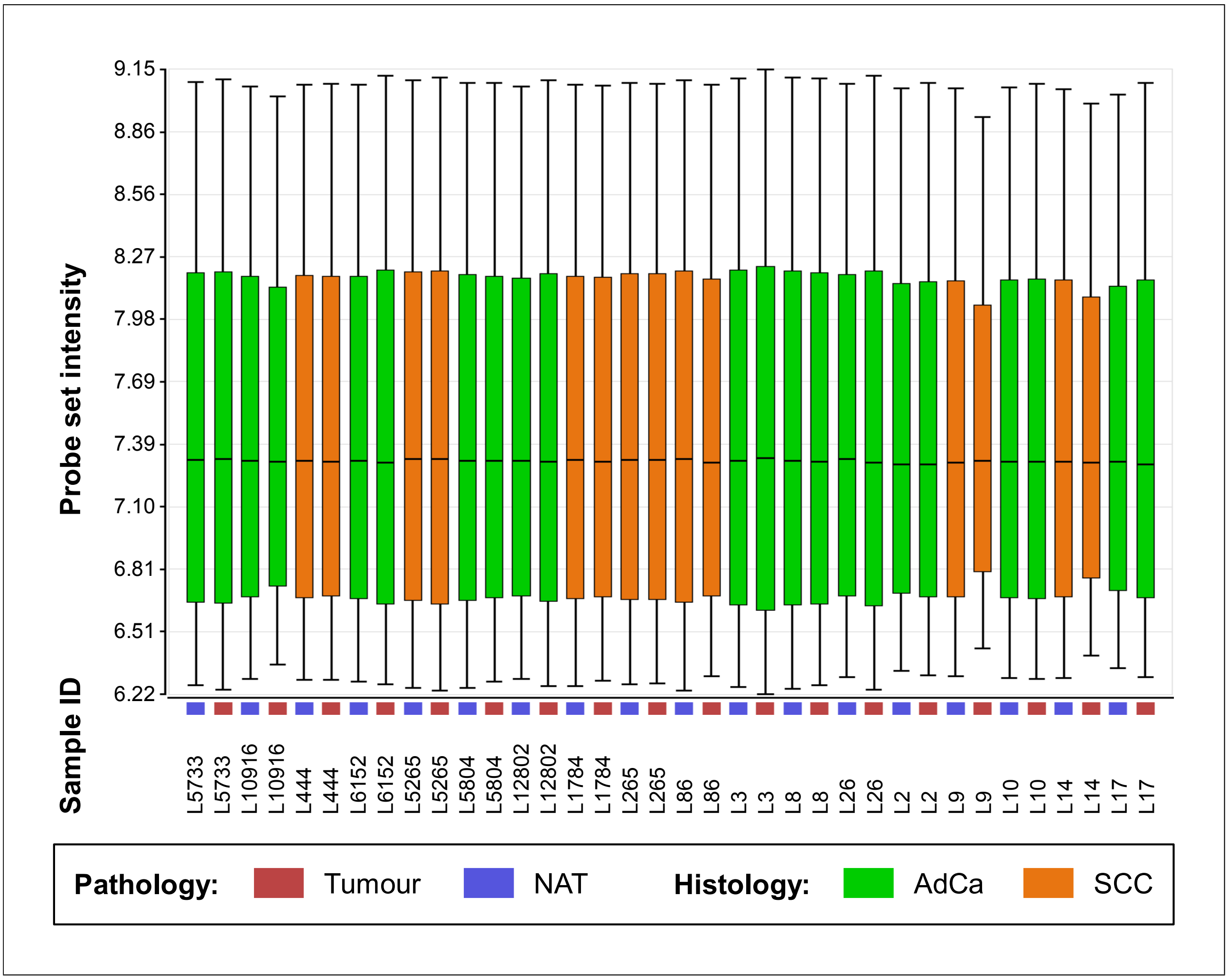

Supplementary figure S5–3: Distributions of log-transformed probe set intensities per sample shown as box plots. Central line: Median. Lower and upper end of the box: First and third quartiles. Lower and upper whiskers: 10 %- and 90 %-quantiles.

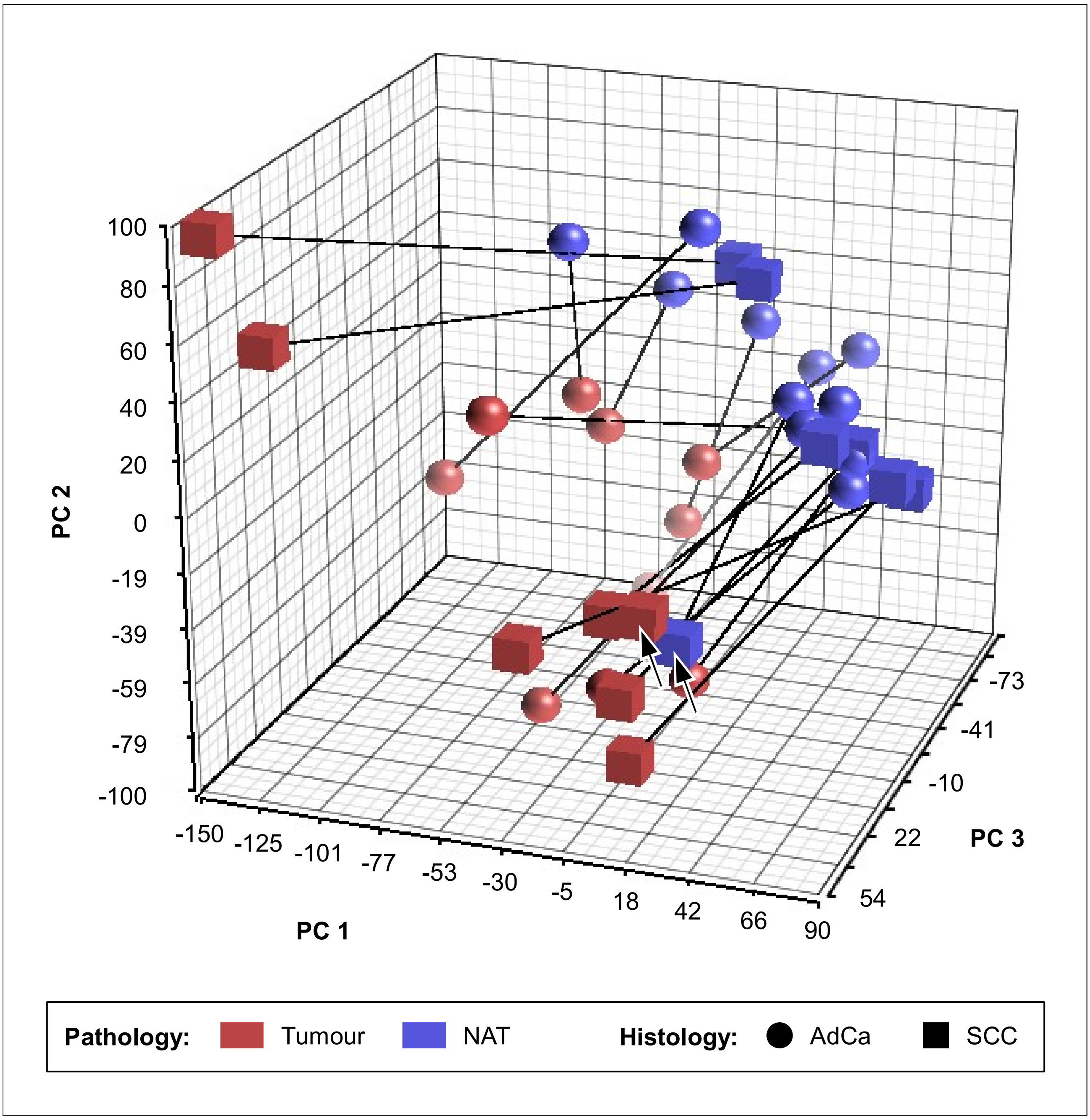

Supplementary figure S5–4: Principal component analysis of gene level intensities. The first, second, and third principal components (PC) are shown in a 3D-scatter plot. Paired samples of a patient are connected with a line. Patient L1784 is located in the centre (highlighted by arrows). Tumour samples of the patients L9 and L14 are located in the top-front-left octant.

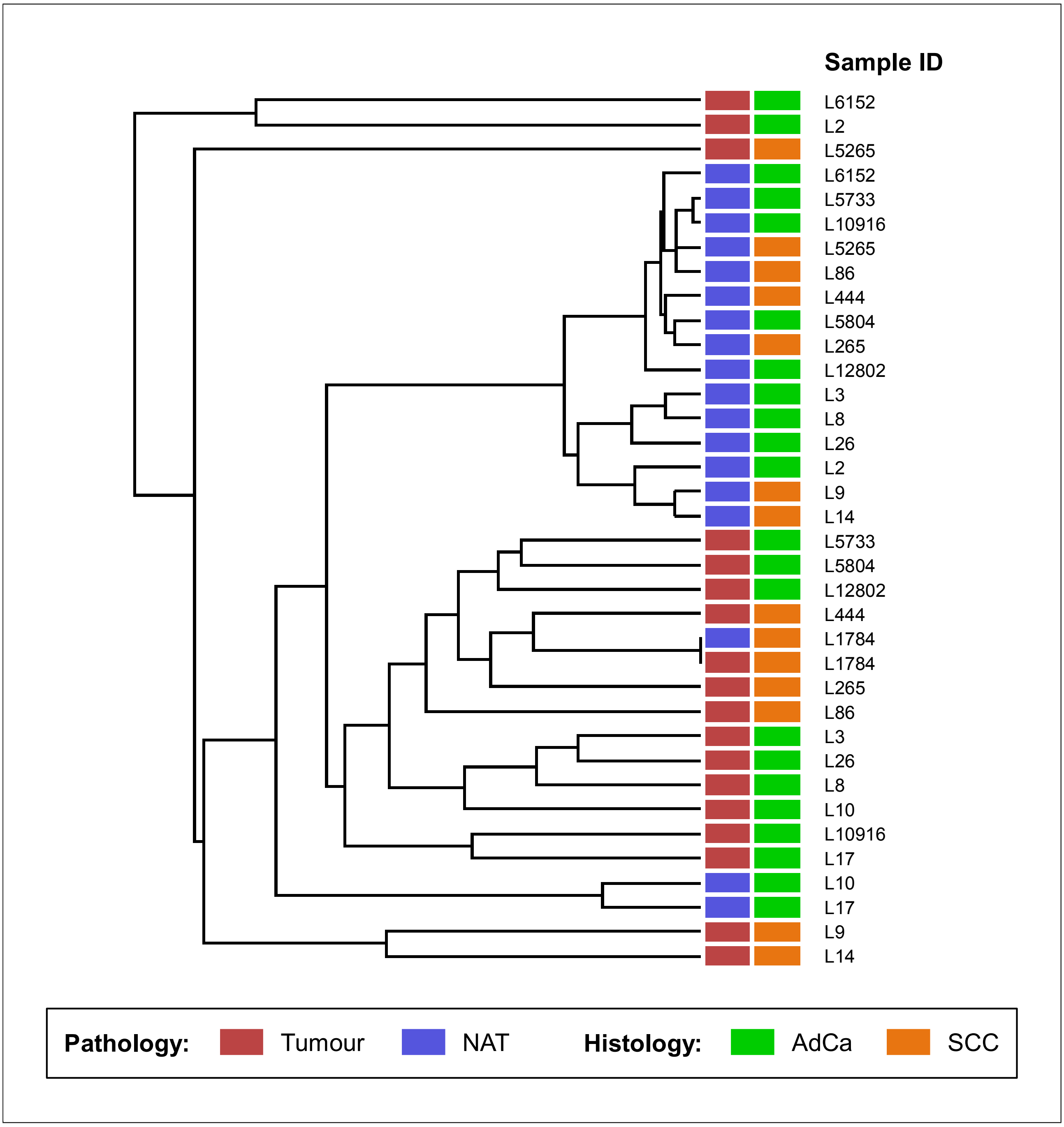

Supplementary figure S5–5: Unsupervised hierarchical clustering of gene level intensities. Pearson's dissimilarity metric, average linkage.
