# Supplementary material for: Exon Array Analysis using re-defined probe sets results in reliable identification of alternatively spliced genes in non-small cell lung cancer
Source: BMC Genomics. 2010 Nov 30;11:676. doi: 10.1186/1471-2164-11-676 (PMC3053589; doi:10.1186/1471-2164-11-676)
Supplement: Additional file 11 — Table S11: Validation results of candidate genes. [file 1471-2164-11-676-S11.PDF]

Supplementary table S11: Validation results of candidate genes.

Gene	Exon array gene profile	RT-PCR	Sequencing	qRT-PCR
ANTXR1<br>Alternative transcription start site	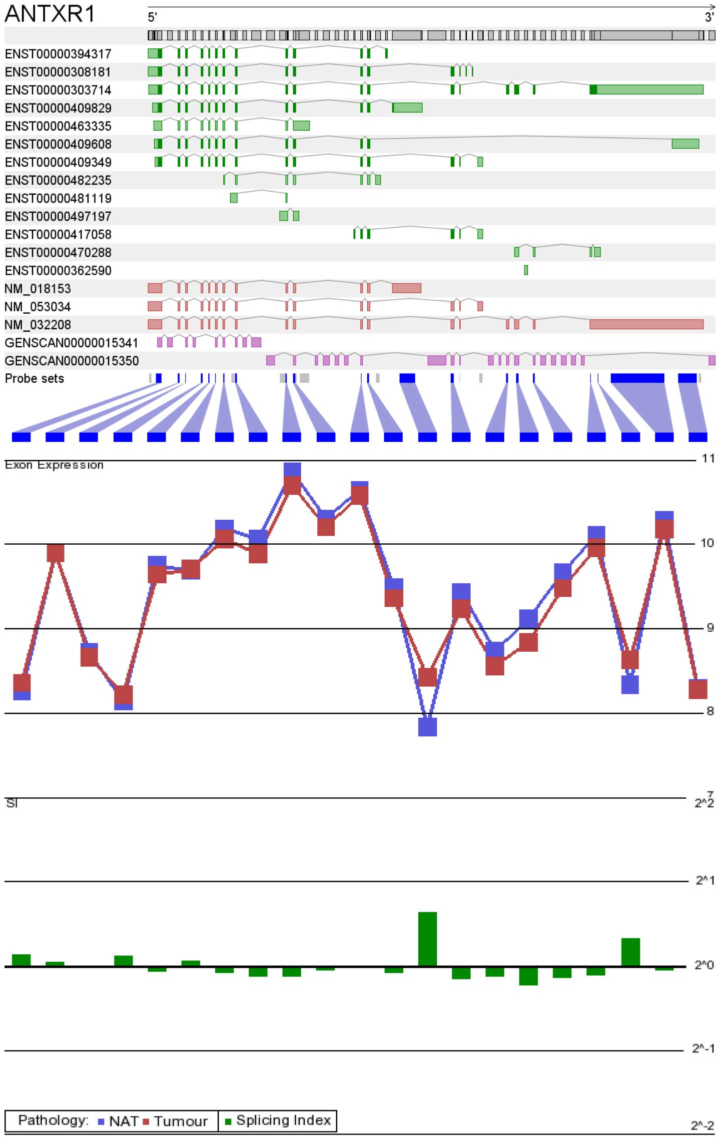	N/A	N/A	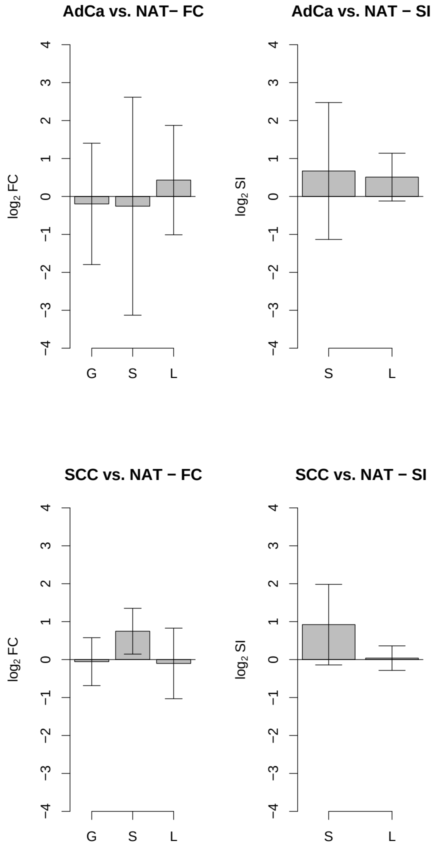

Supplementary table S11: continued

Gene	Exon array expression profile	RT-PCR	Sequencing	qRT-PCR
CEACAM1 Cassette exon	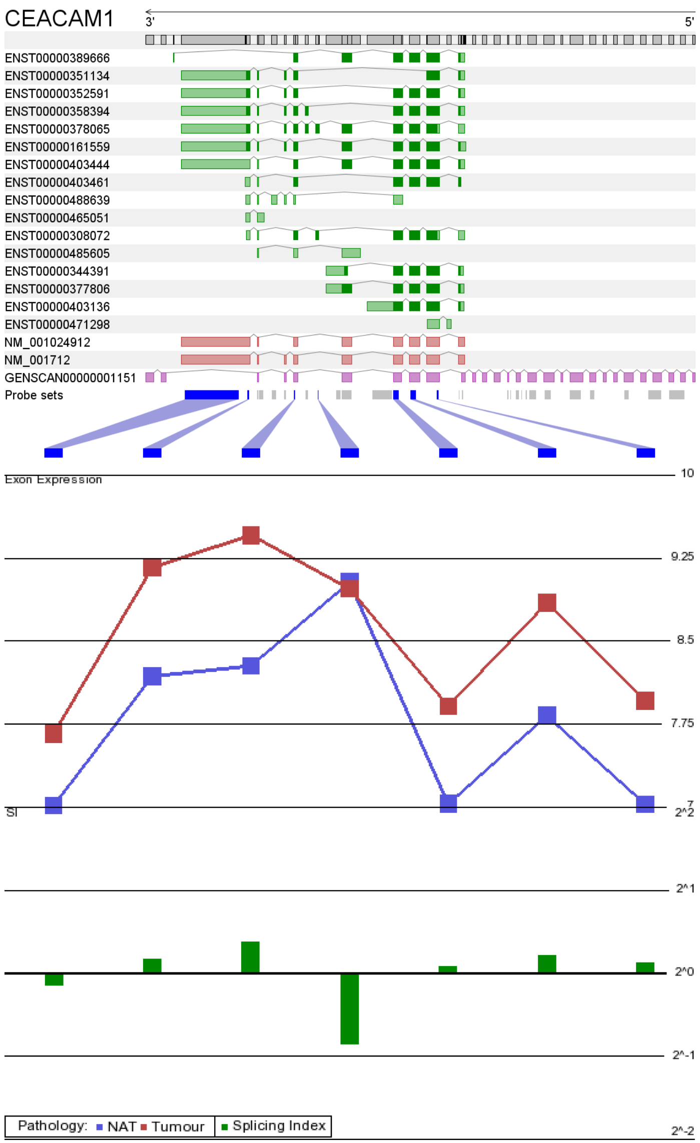	N/A	N/A	N/A

Supplementary table S11: continued

Gene	Exon array expression profile	RT-PCR	Sequencing	qRT-PCR
CLSTN1 Cassette exon	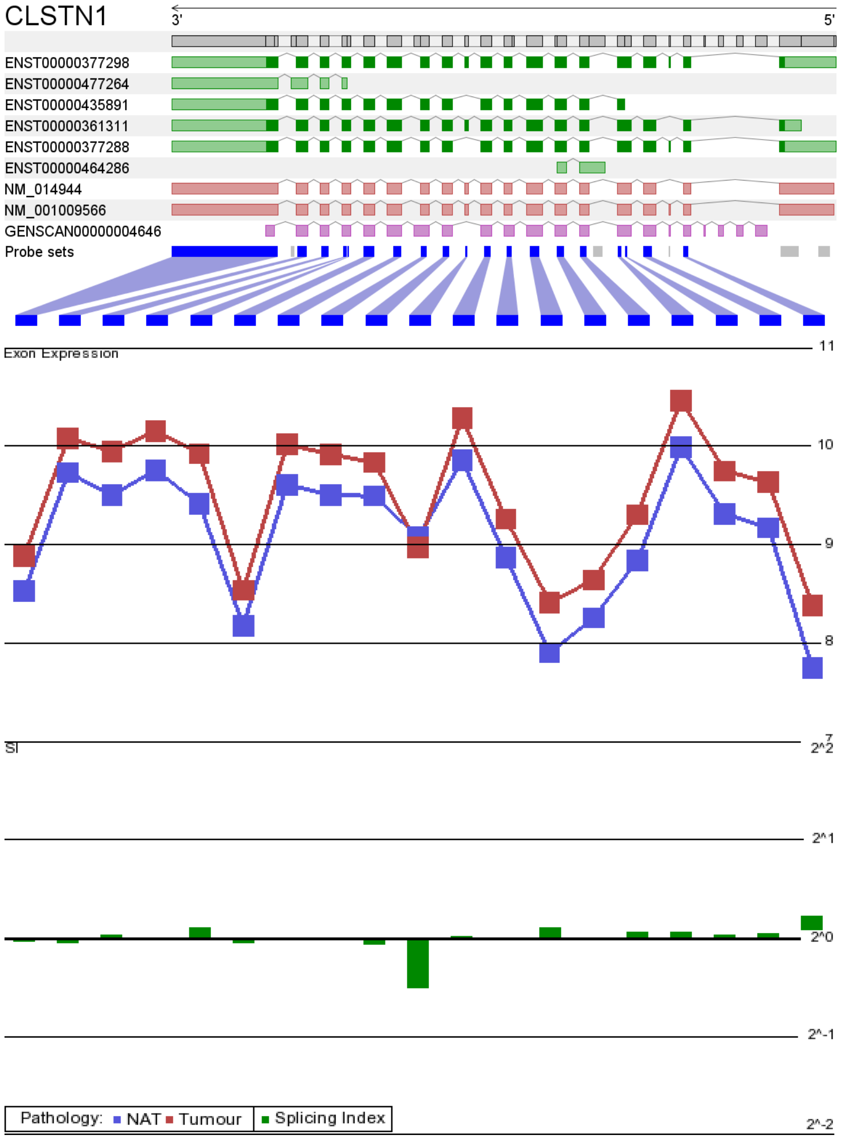	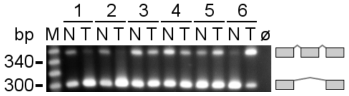	+	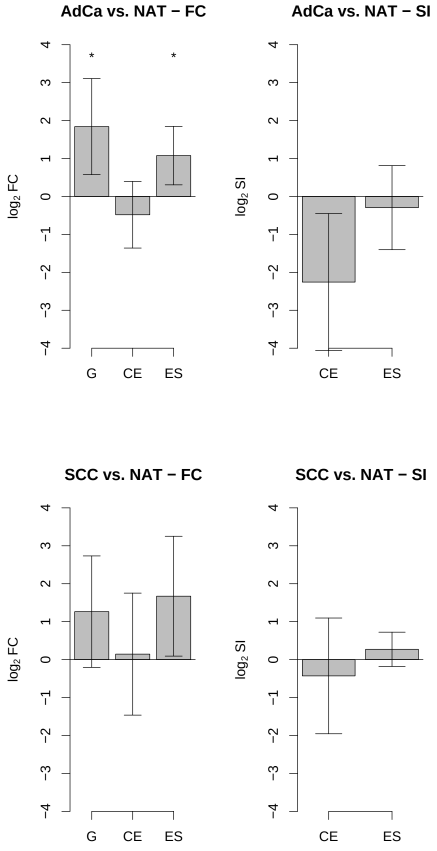

Supplementary table S11: continued

Gene	Exon array expression profile	RT-PCR	Sequencing	qRT-PCR
CTNND1 Cassette exon (5')	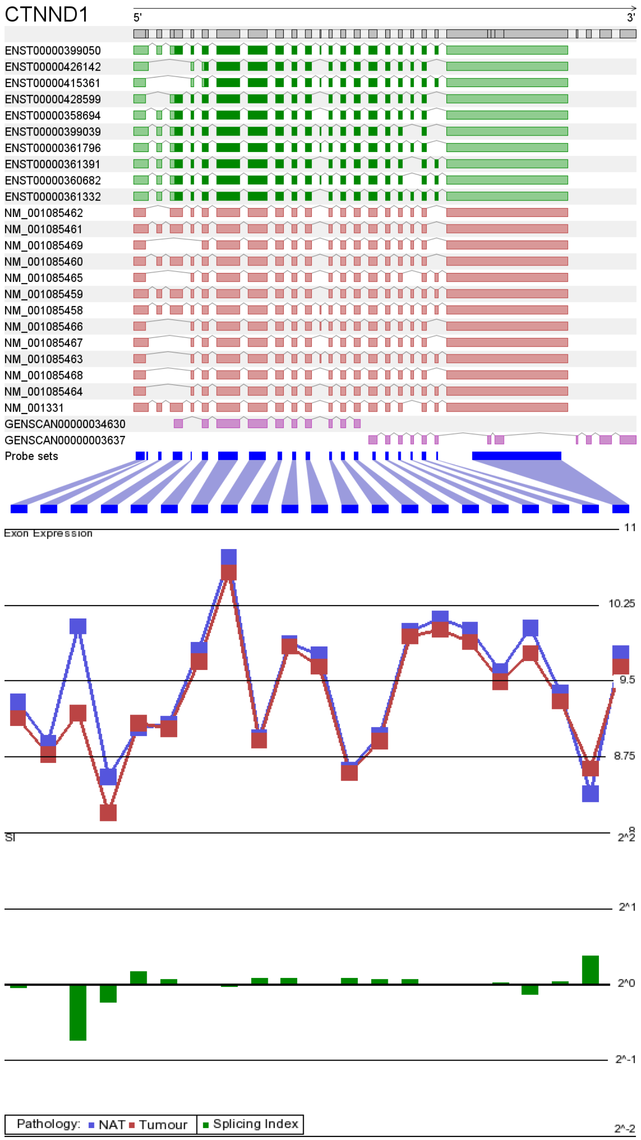	–	N/A	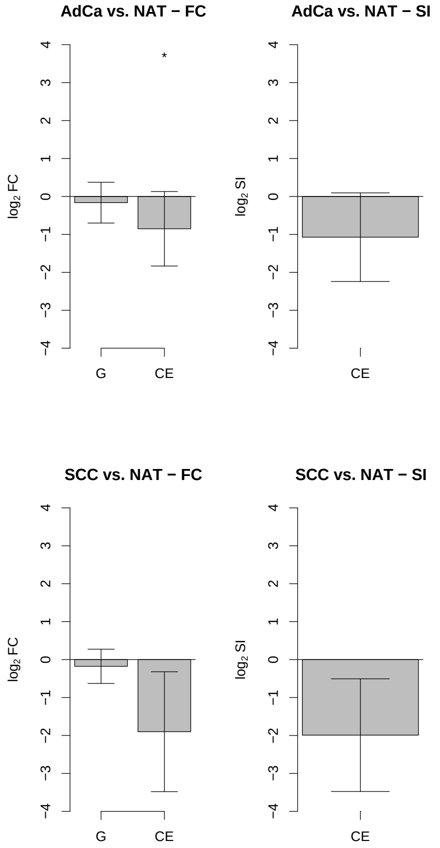

Supplementary table S11: continued

Gene	Exon array expression profile	RT-PCR	Sequencing	qRT-PCR
CTNND1 Cassette exon (3')	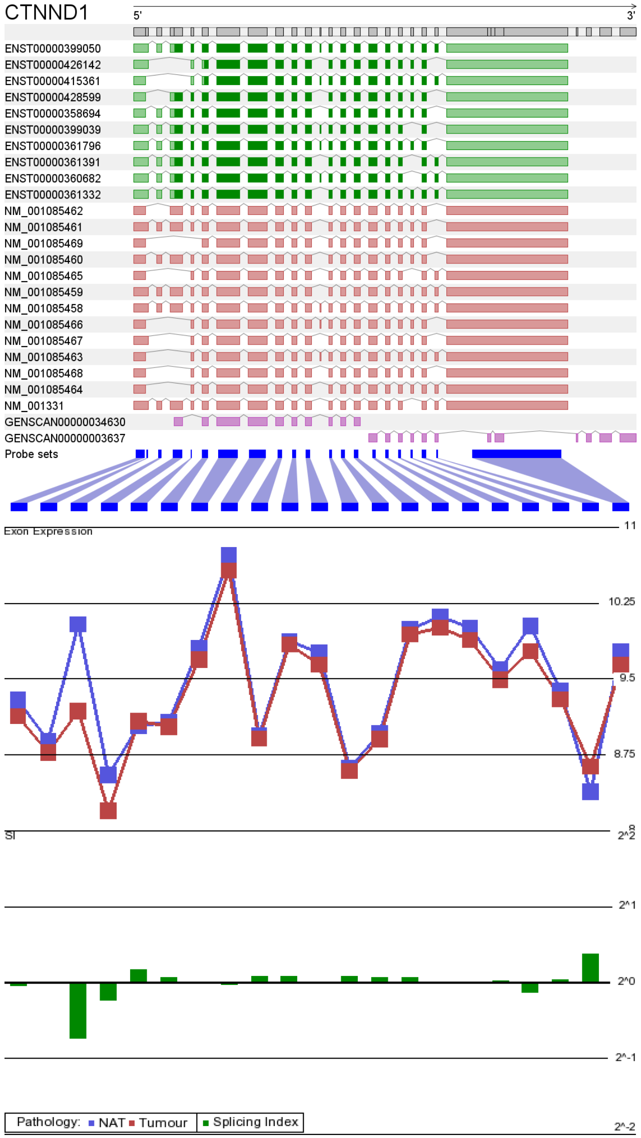	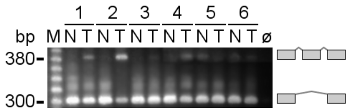	+	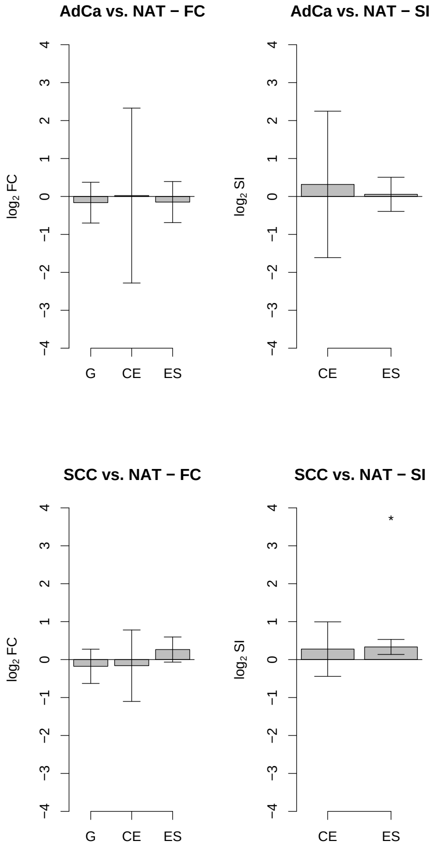

Supplementary table S11: continued

Gene	Exon array expression profile	RT-PCR	Sequencing	qRT-PCR
FN1 Cassette exon	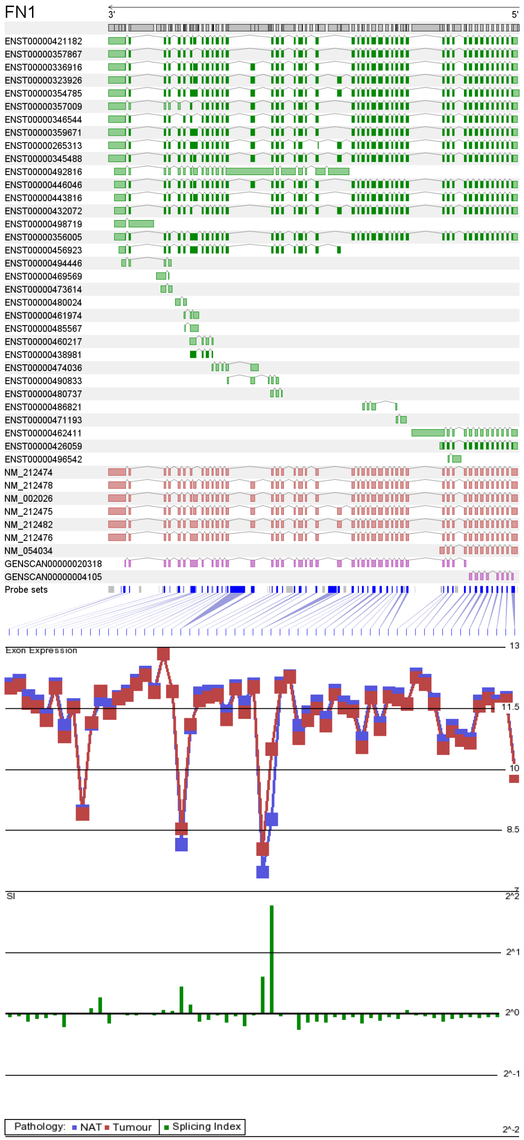	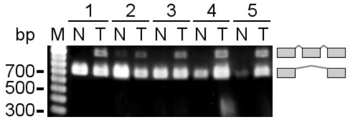	+	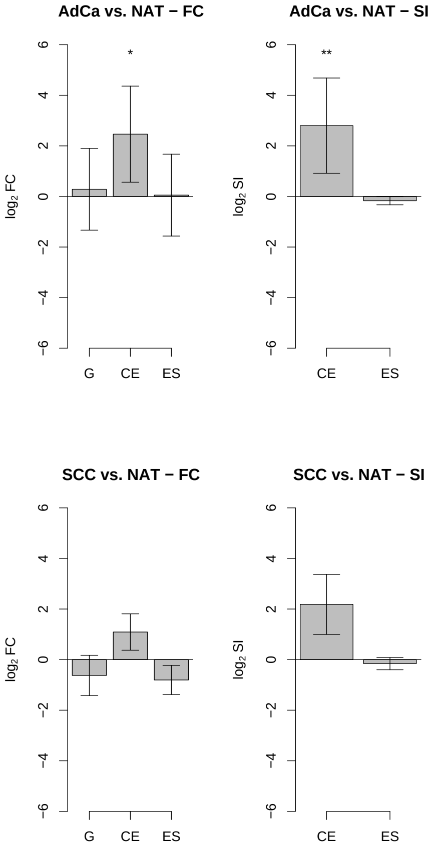

Supplementary table S11: continued

Gene	Exon array expression profile	RT-PCR	Sequencing	qRT-PCR
KIAA1217 Alternative transcription start site	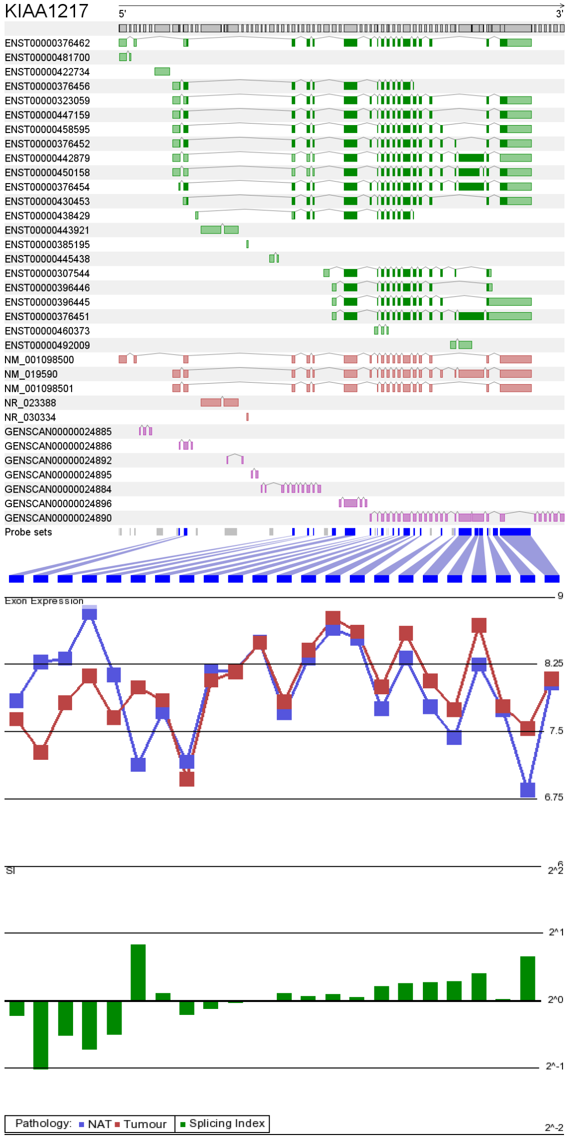	N/A	N/A	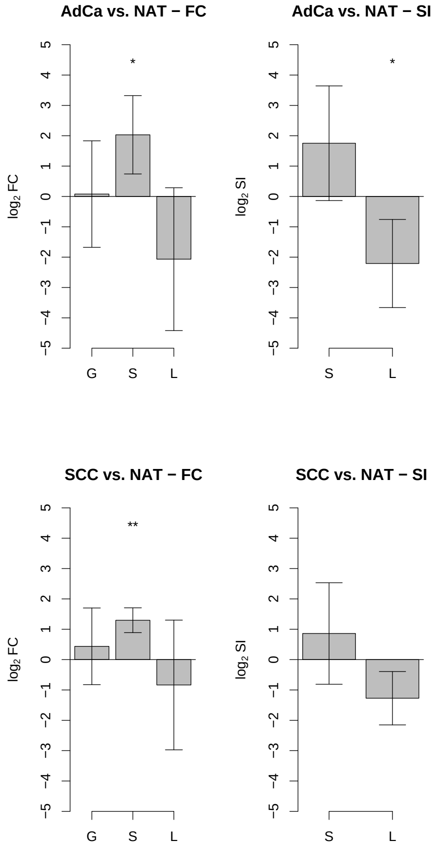

Supplementary table S11: continued

Gene	Exon array expression profile	RT-PCR	Sequencing	qRT-PCR
KIAA1217<br>Intron reten-<br>tion	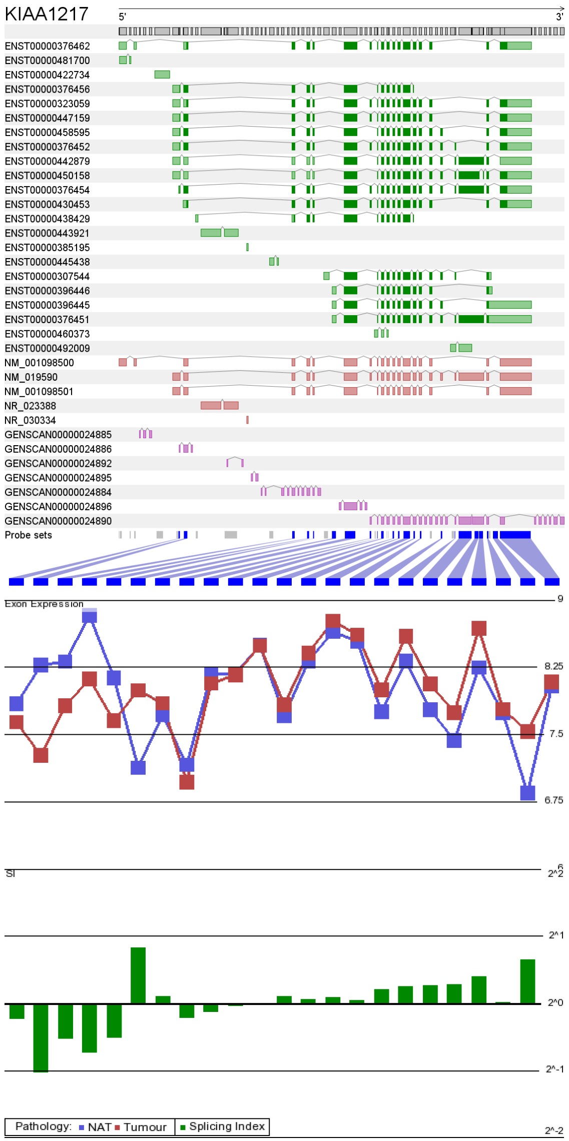	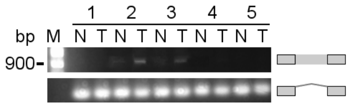	+	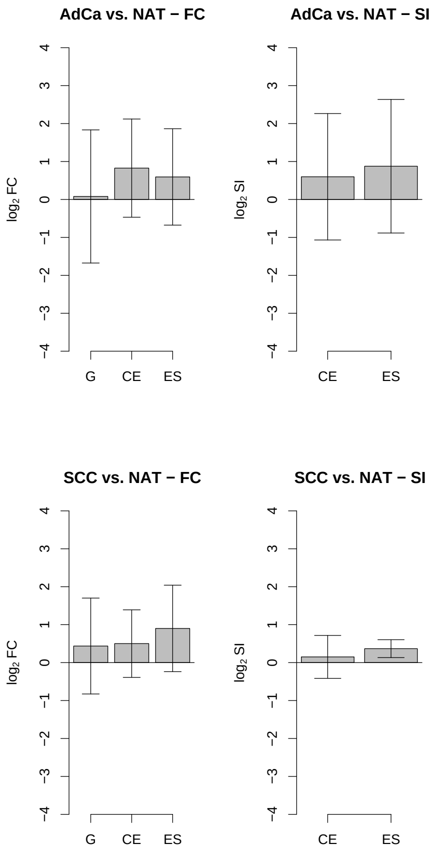

Gene	Exon array expression profile	RT-PCR	Sequencing	qRT-PCR
MYH14 Cassette exon	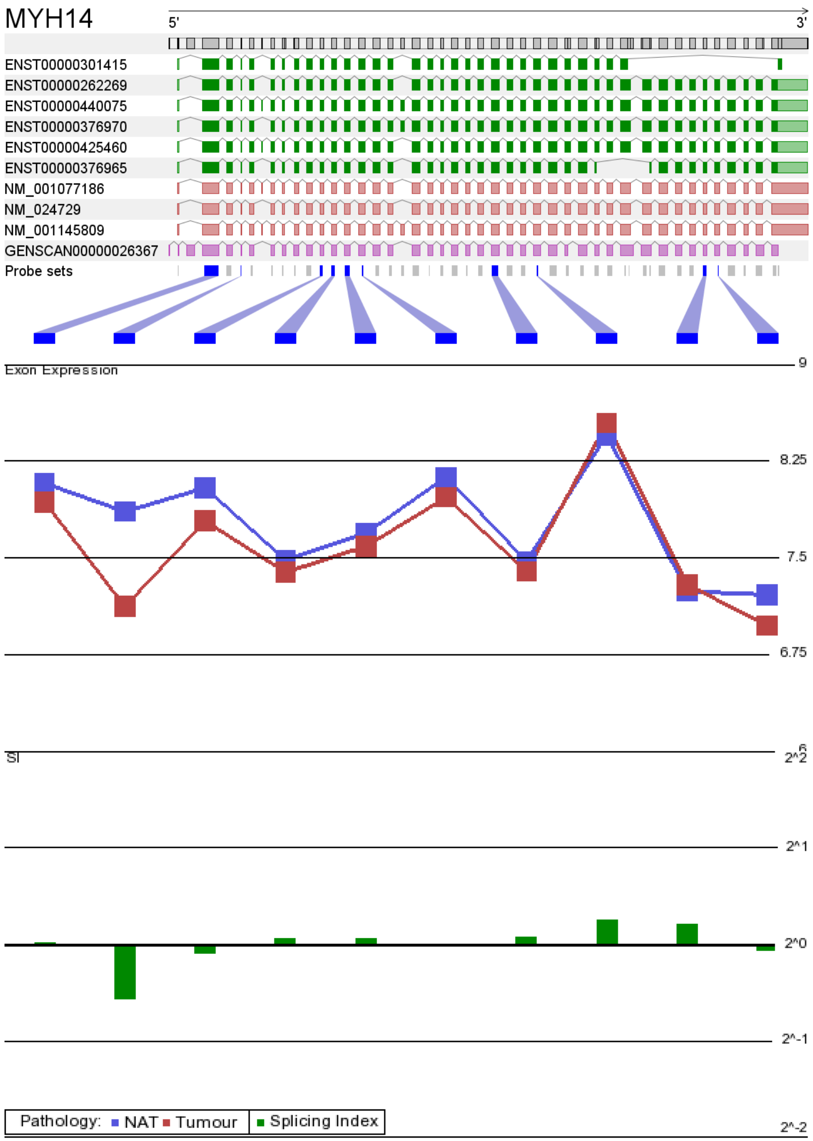	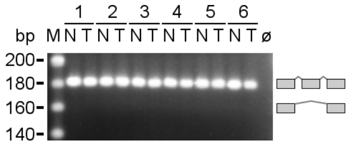	+	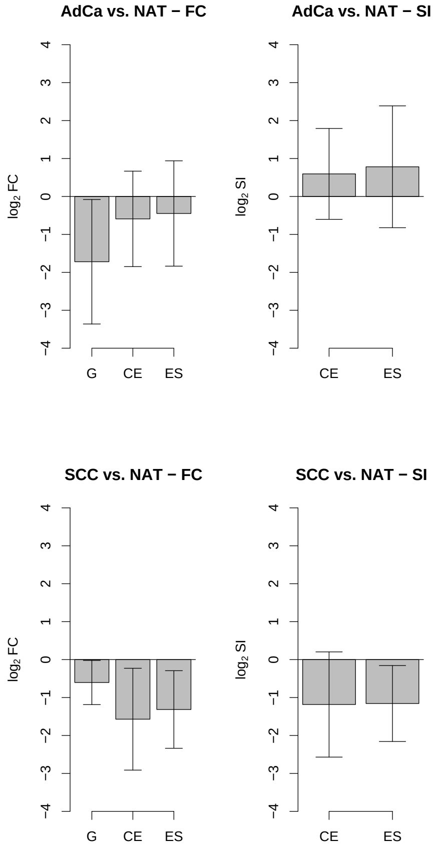

Gene	Exon array expression profile	RT-PCR	Sequencing	qRT-PCR
MYO18A Cassette exon	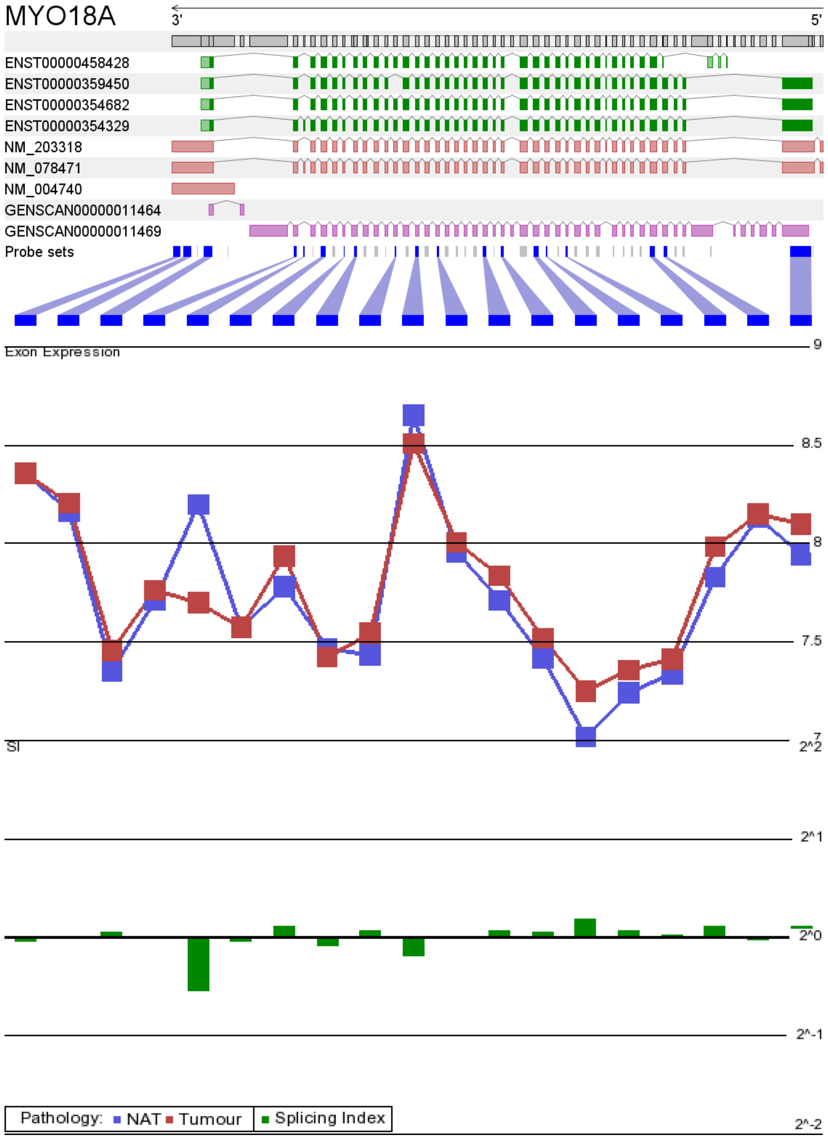	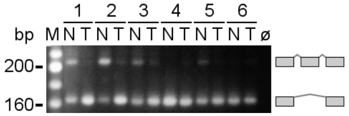	+	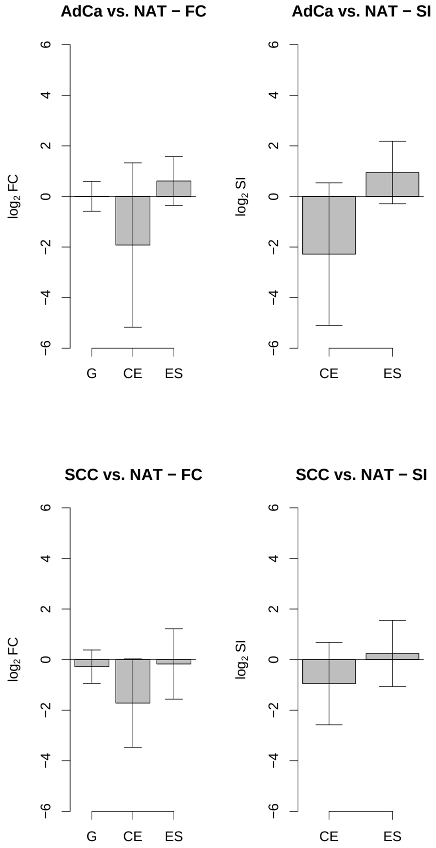

<table>
<tr><th>Gene</th><th>Exon array expression profile</th><th>RT-PCR</th><th>Sequencing</th><th>qRT-PCR</th></tr>
<tr><td>NUMB Cassette exon</td><td>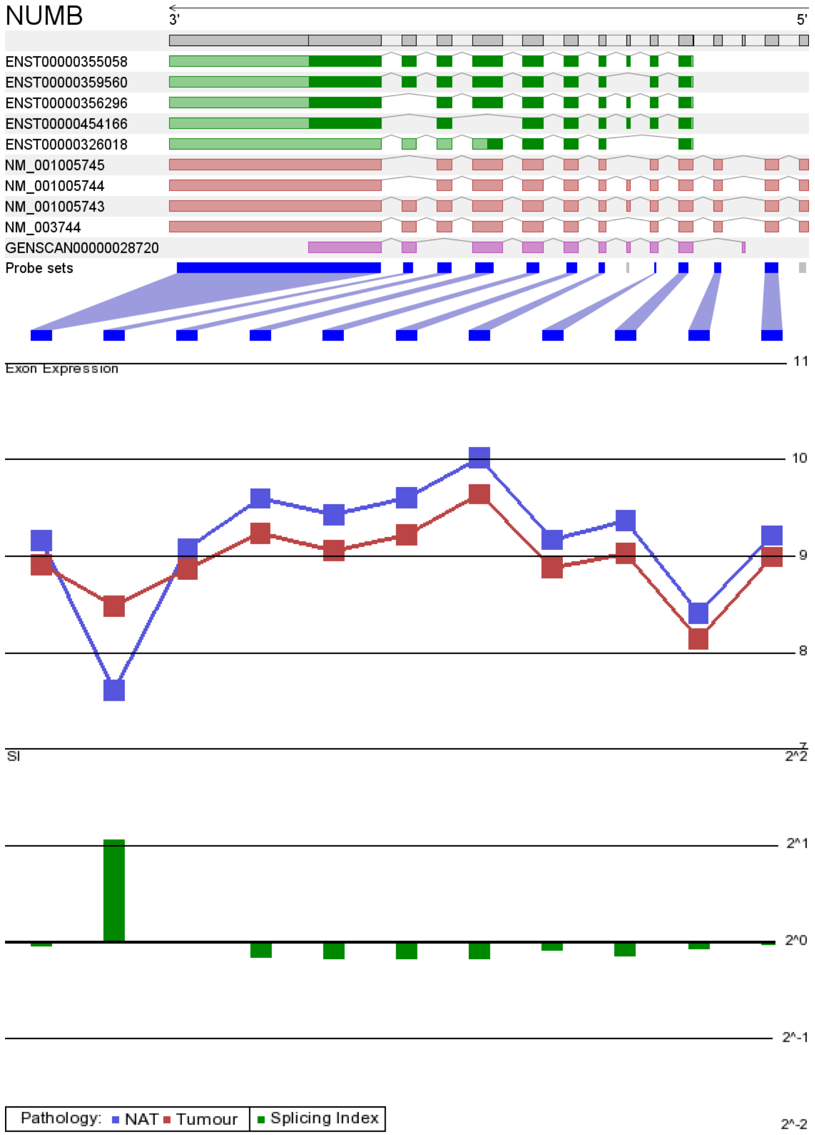
</td><td>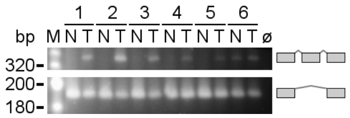
</td><td>+</td><td>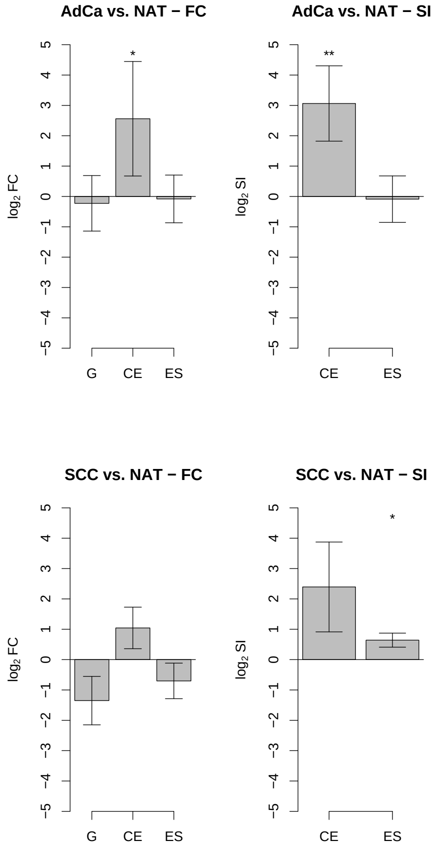
</td></tr>
</table>

Gene	Exon array expression profile	RT-PCR	Sequencing	qRT-PCR
SLK Cassette exon	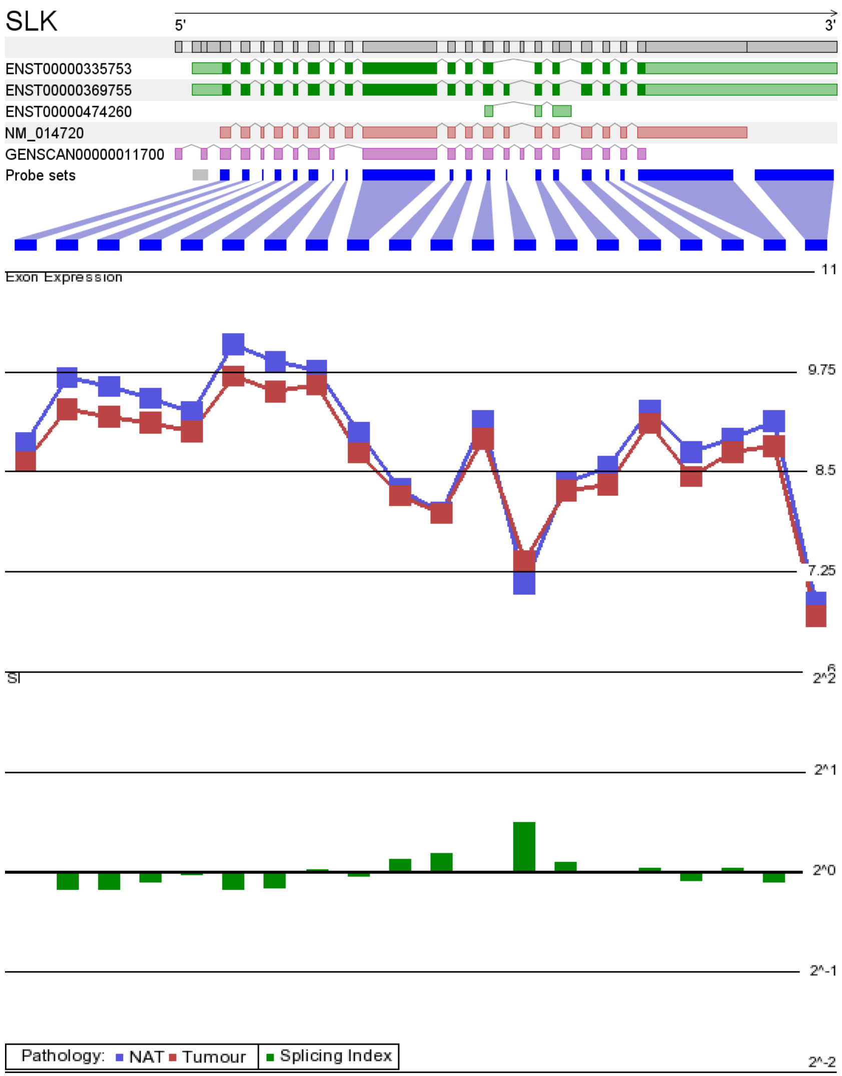	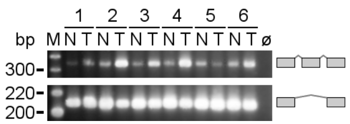	+	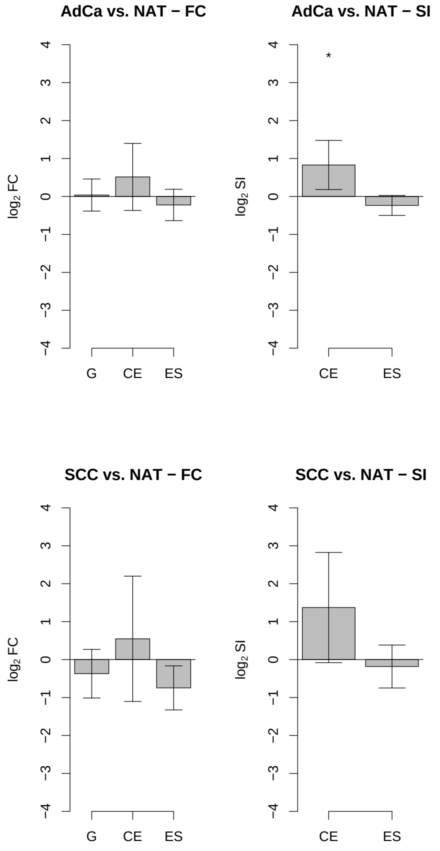

Supplementary table S11: continued

Gene	Exon array expression profile	RT-PCR	Sequencing	qRT-PCR
SYNE2 Cassette exon	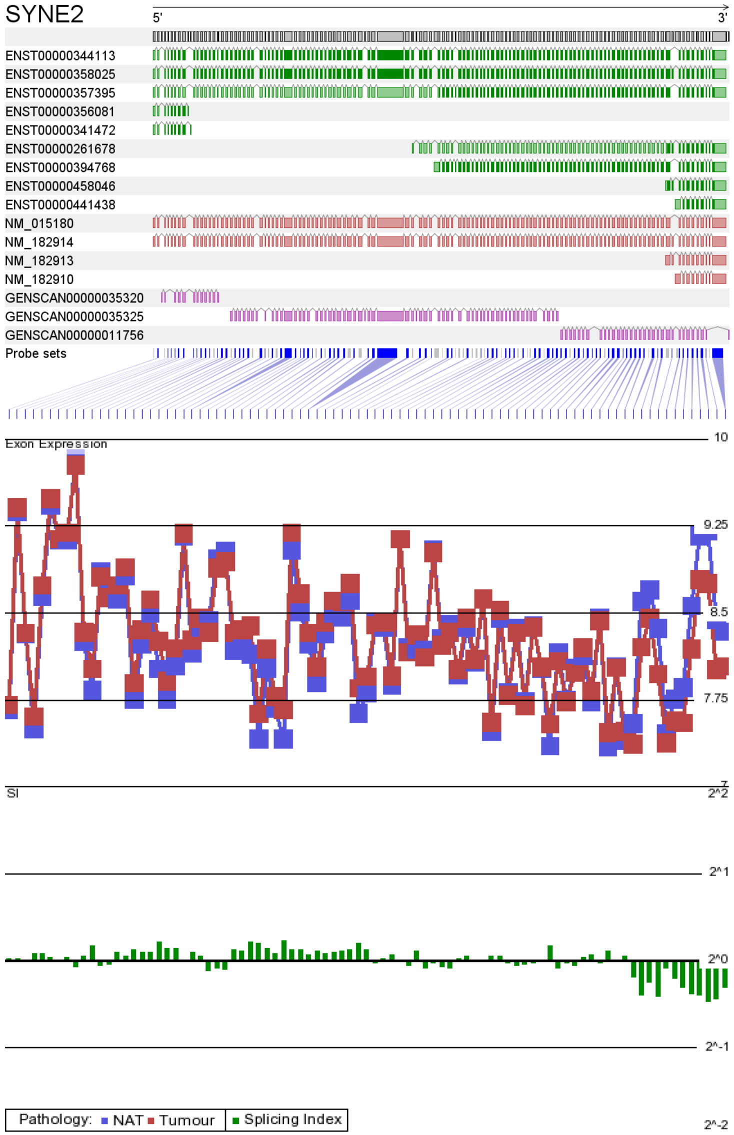	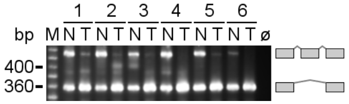	+	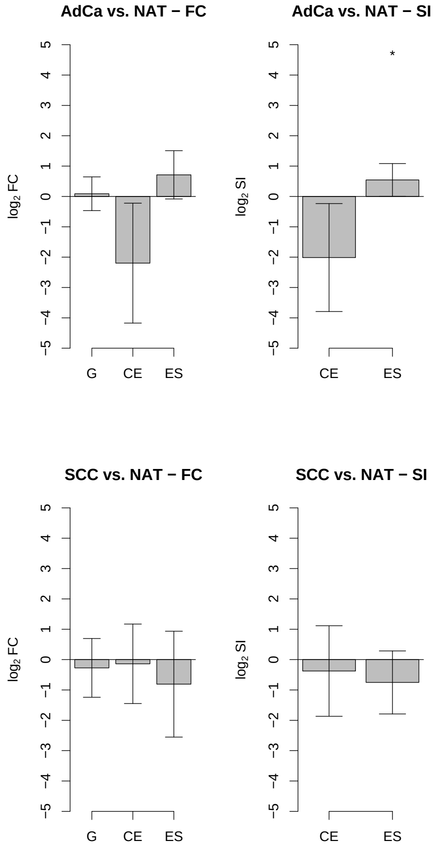

Supplementary table S11: continued

Gene	Exon array expression profile	RT-PCR	Sequencing	qRT-PCR
TPM1 Mutually exclusive exon (5')	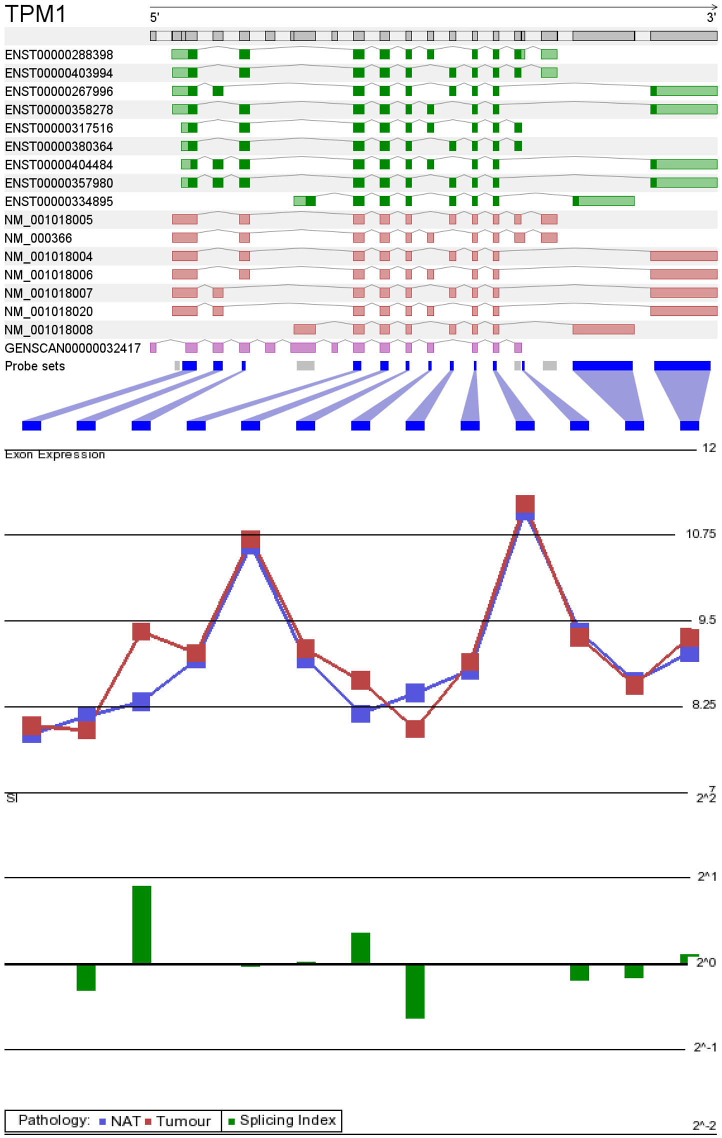	N/A	+	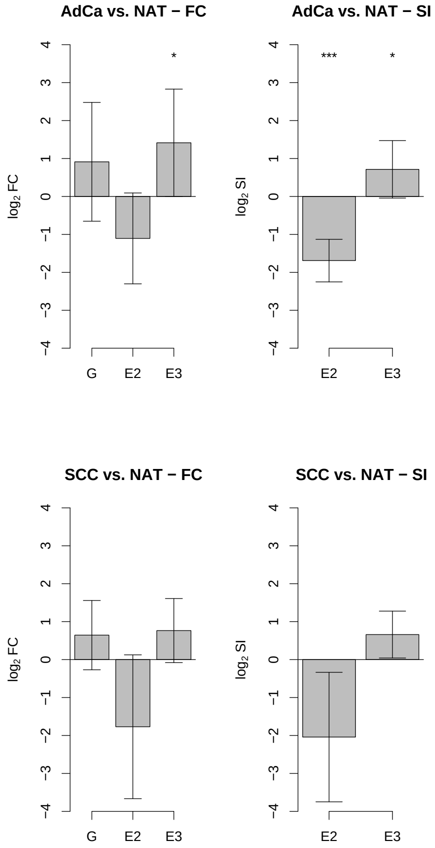

Supplementary table S11: continued

Gene	Exon array expression profile	RT-PCR	Sequencing	qRT-PCR
TPM1 Mutually exclusive exon (3')	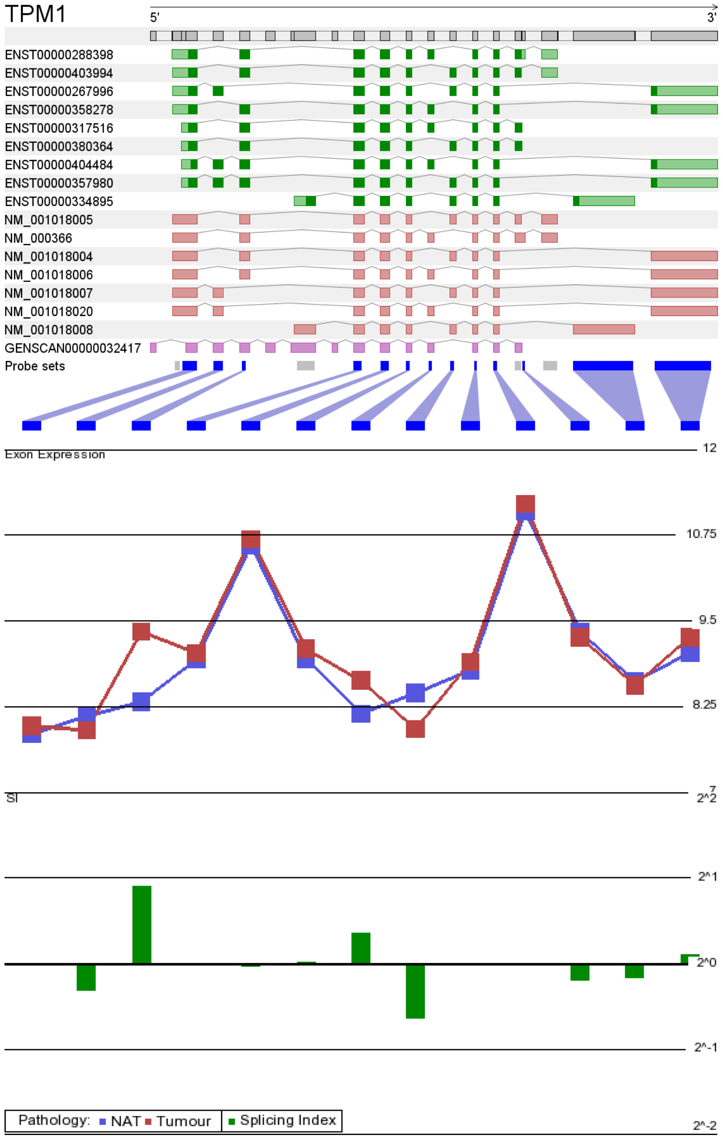	N/A	+	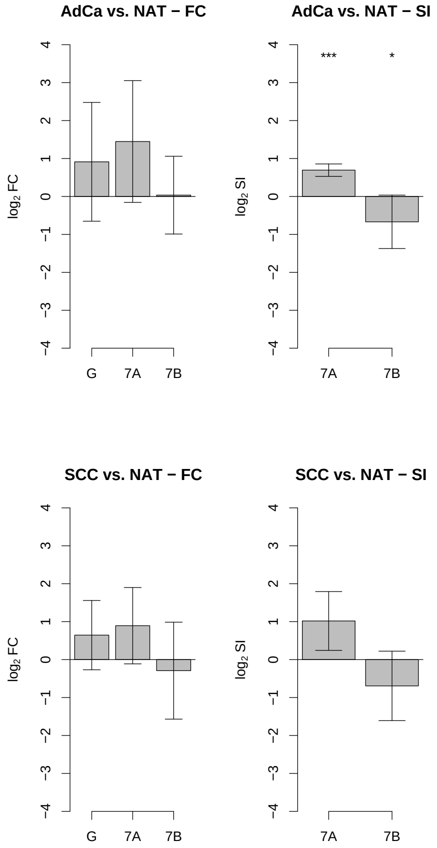

The exon array gene profile is shown together with the exon structure of the gene (introns not to scale), known transcript annotations (green: Ensembl transcripts; red: RefSeq transcripts; purple: Genscan predictions), and exon array probe sets from the new chip definition (grey: absent probe sets; blue: present probe sets). The exon expression in the NSCLC data set (red graph: exon expression in NSCLC; blue graph: exon expression in normal adjacent tissue) as well as splicing indexes for exons in the NSCLC data set (green bars; logarithmic scale) are shown. A hypothesis of the mode of alternative splicing was formulated based on the exon array expression profile and available transcript annotations. RT-PCR was conducted using paired samples of adenocarcinoma of NSCLC and normal adjacent tissue of six patients (ø: no template control). Assays were designed such that primer pairs are flanking a cassette exon, thus alternative splicing generates two RT-PCR products. Exon-exon junctions in RT-PCR products were confirmed by sequencing (data not shown). qRT-PCR was performed using six sample pairs of adenocarcinoma of NSCLC and four sample pairs of squamous cell carcinoma of NSCLC. Assays were designed to measure gene level expression (G) or to measure a specific transcript variant (CE: cassette exon; ES: exon skipping; S: short isoform; L: long isoform; IR: intron retention; IS: intron skipping). Error bars indicate one standard deviation, significance was determined using a paired t-test. FC: Fold-change of over-expression in NSCLC versus normal adjacent tissue. SI: Splicing index. N/A indicates that the experiment was not performed.
