# Supplementary material for: Exon Array Analysis using re-defined probe sets results in reliable identification of alternatively spliced genes in non-small cell lung cancer
Source: BMC Genomics. 2010 Nov 30;11:676. doi: 10.1186/1471-2164-11-676 (PMC3053589; doi:10.1186/1471-2164-11-676)
Supplement: Additional file 12 — Text S12: Quantification of NCOR2 transcript variants in NSCLC. [file 1471-2164-11-676-S12.PDF]

S12: Quantification of NCOR2 transcript variants in NSCLC

NCOR2 is a gene that contains at least 47 exons (Supplementary fig. S12–1a). One exon, near the 3'-end, contains an internal alternative 5'-splice site that can generate a shortened form of this exon. In Ensembl the long exon of 225 bp is designated as ENSE00000939878 and the shorter form of 87 bp as ENSE00001528376. Probes on the exon array covered both tails of the exon so that two probe sets were defined (Supplementary fig. S12–1b). Results from the NSCLC exon array data set show a high SI for the longer isoform of NCOR2 (Supplementary fig. S12–1c-d). This suggests that the internal splice site is used predominantly in NAT, whereas the longer form of the exon can be found in tumours. This differential splicing pattern was observed in 14 of 18 patients (78 %). Three patients showed an inverse pattern and one patient did not exhibit any indication of differential splicing. These patients are not the same as those displaying exceptional behaviour in ADD3. Neither is there a correlation with factors such as subtype or staging.

RT-PCR results generated using AdCa samples reveal that both transcript variants of NCOR2 are equally expressed in normal lung tissue of most patients (Supplementary fig. S12–1e). In tumour, however, there is a shift towards the longer transcript variant, i.e. splicing does not involve the internal splice site. In two patients, the pattern was different. One patient did not exhibit the shift; thus, there is no difference between AdCa and NAT. Neither does the exon array result of this patient correspond with the major trend. Yet another patient exhibited an inverse pattern in the RT-PCR result. Here, the exon array result contradicts the RT-PCR result.

Total gene expression of NCOR2 and expression of both transcript variants were quantified using specific primer pairs in a qRT-PCR (Supplementary fig. S12–1f-g). Both in AdCa and SCC, the overall expression of NCOR2 seems to be slightly reduced compared to NAT. Therefore, we normalised the transcript variant expression values by calculating the SI for each patient individually. It became evident that there is a significant increase of the long isoform in AdCa versus NAT. On average, expression of the short isoform remains unchanged. The picture is different for SCC: here, a decreased expression of the long isoform and an increased expression of the short isoform was observed; however, variance was high. For the average expression of the long isoform, exon array results showed no difference between AdCa and SCC; thus, the qRT-PCR result contradicts the exon array result. An analysis of SI values per patient individually revealed a strong increase for one patient, for another, a strong decrease, and for two patients, a less pronounced decrease of expression of the long transcript variant. Both of the extreme changes were also evident in the exon array results, whereas the smaller changes could not be confirmed.

All results indicate that there is a shift in the alternative splicing pattern of NCOR2 in tumour versus NAT. At least in AdCa, the long isoform of NCOR2 is preferred. Significance has not been reached for the SCC samples and with the given power results for this subtype are inconclusive.

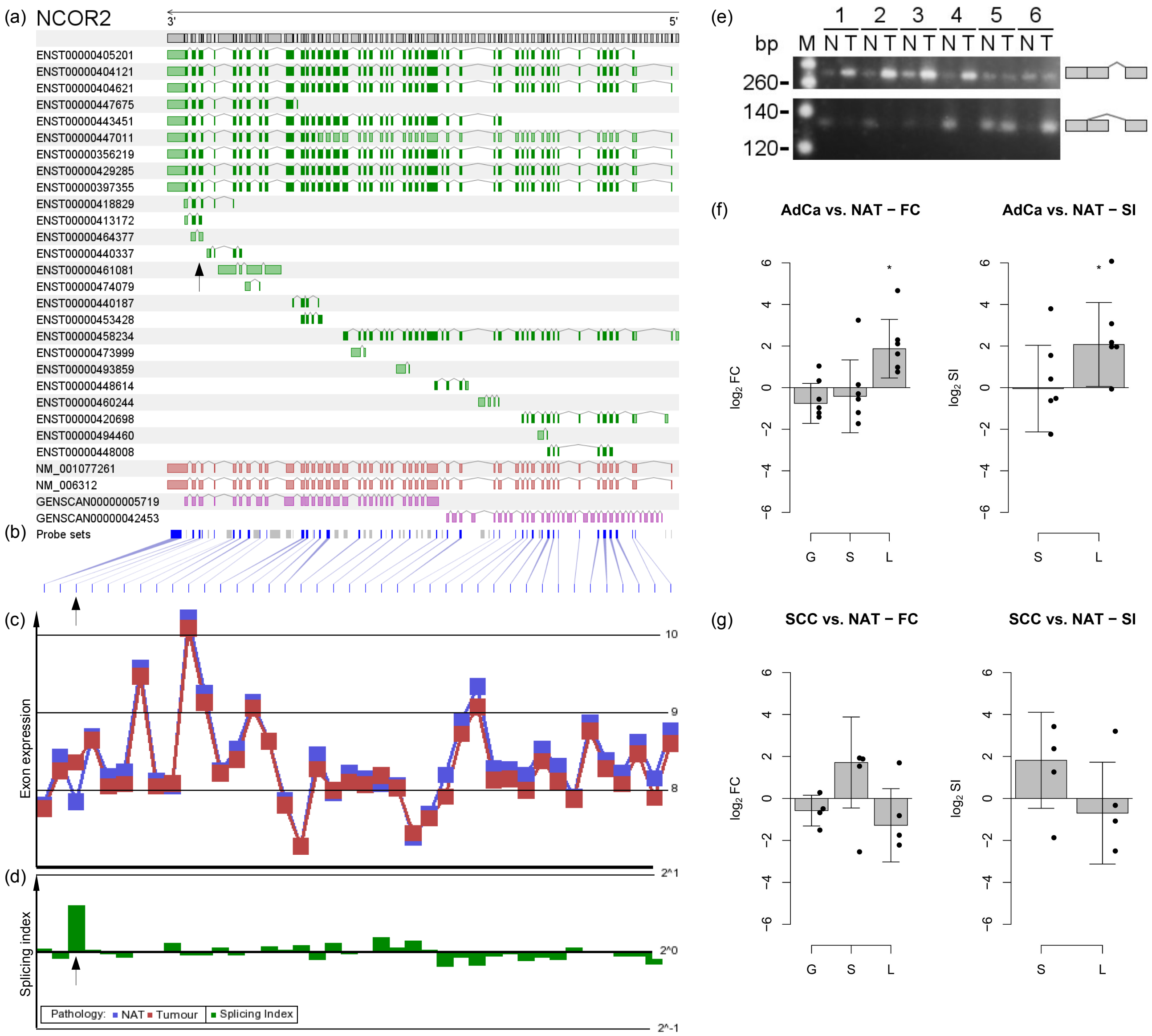

Supplementary figure S12–1: Details of the exon array results and the laboratory validation results for NCOR2. **(a)** Exon structure and known transcript variants of NCOR2 (introns not to scale; green: Ensembl transcripts; red: RefSeq entries; purple: Genscan predictions). **(b)** Position of probe sets in the new exon array chip definition (grey: absent probe sets; blue: present probe sets). **(c)** Exon expression in the NSCLC data set suggests a prolonged exon (arrow) in tumour compared to normal adjacent tissue (NAT) (red graph: exon expression in NSCLC; blue graph: exon expression in NAT). **(d)** Splicing indices for exons in the NSCLC data set (logarithmic scale). **(e)** Verification of RT-PCR product sizes generated from paired samples of adenocarcinoma of NSCLC and normal adjacent tissue of six patients (ø: no template control). In tumour, a shift to the longer exon variant can be observed in four of six cases analysed. Sequencing of representative products confirmed the expected exon-exon junctions (data not shown). **(f)** Quantification of gene expression (G) and transcript variant expression (S: short exon; L: long exon) in adenocarcinoma of NSCLC compared to normal adjacent tissue (NAT) as measured by qRT-PCR. Median values based on six sample pairs are shown (values for each patient shown as dots), error bars indicate one standard deviation, significance was determined using a paired t-test. FC: Fold-change of over-expression in adenocarcinoma of NSCLC versus NAT. SI: Splicing index. **(g)** Quantification of gene expression and transcript variant expression in squamous cell carcinoma of NSCLC compared to NAT as measured by qRT-PCR. Median values based on four sample pairs are shown.
