# Supplementary material for: Exon Array Analysis using re-defined probe sets results in reliable identification of alternatively spliced genes in non-small cell lung cancer
Source: BMC Genomics. 2010 Nov 30;11:676. doi: 10.1186/1471-2164-11-676 (PMC3053589; doi:10.1186/1471-2164-11-676)
Supplement: Additional file 14 — Figure S14: Expression of FOX2 in twelve different types of cancer and corresponding normal tissue as well as in 50 other healthy tissues. Geometric mean signal intensities of probe set 212104_s_at (Affymetrix expression array HG-U133_Plus_2.0) which measures gene expression of RBM9 (FOX2). The number of samples per group is shown (in total, 1015 samples). Error bars represent one standard deviation as calculated from the log-transformed intensities. [file 1471-2164-11-676-S14.PDF]

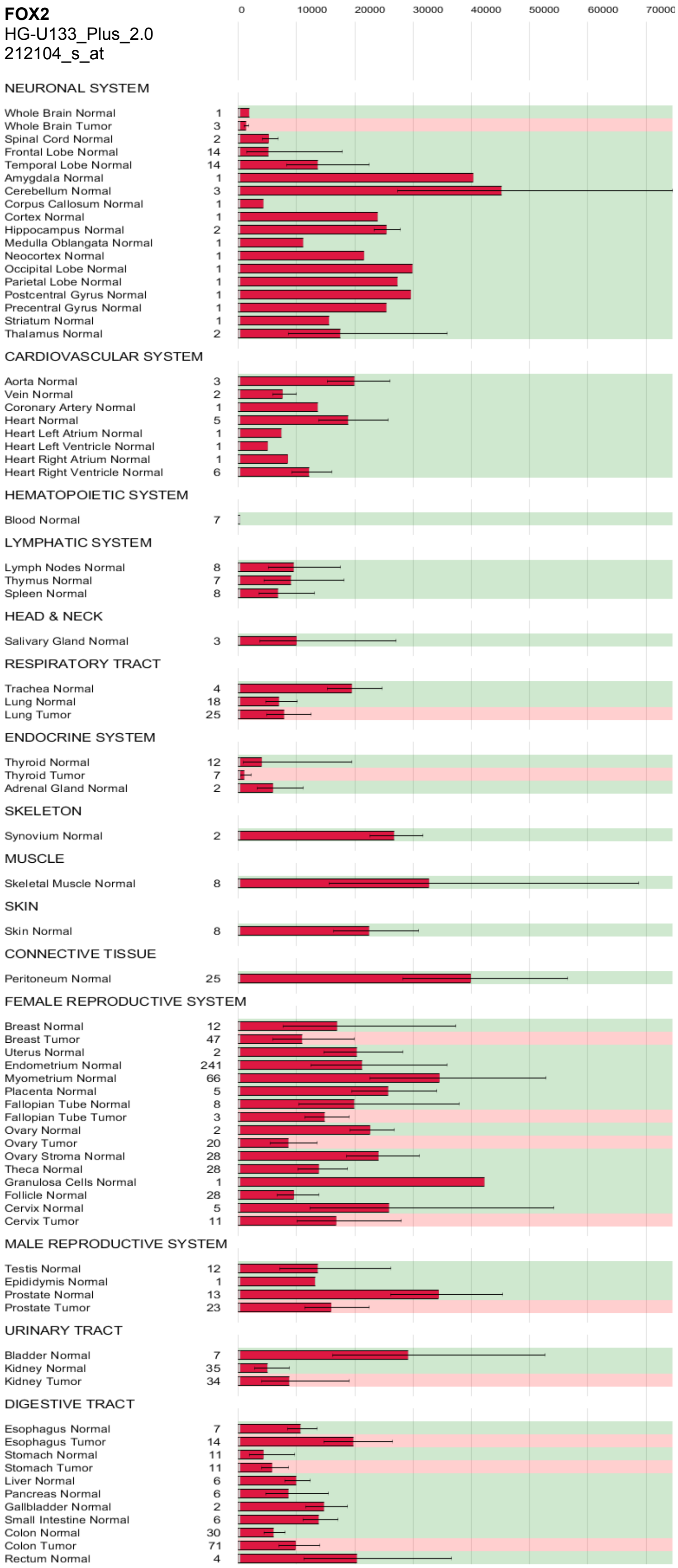

Supplementary figure S14: Expression of FOX2 in twelve different types of cancer and corresponding normal tissue as well as in 50 other healthy tissues. Geometric mean signal intensities of probe set 212104_s_at (Affymetrix expression array HG-U133_Plus_2.0) which measures gene expression of *RBM9* (FOX2). The number of samples per group is shown (in total, 1,015 samples). Error bars represent one standard deviation as calculated from the log-transformed intensities.
